# A new human-inspired metaheuristic algorithm for solving optimization problems based on mimicking sewing training

**DOI:** 10.1038/s41598-022-22458-9

**Published:** 2022-10-17

**Authors:** Mohammad Dehghani, Eva Trojovská, Tomáš Zuščák

**Affiliations:** Department of Mathematics, Faculty of Science, University of Hradec Králové, Rokitanského 62, 500 03 Hradec Králové, Czech Republic

**Keywords:** Engineering, Mathematics and computing

## Abstract

This paper introduces a new human-based metaheuristic algorithm called Sewing Training-Based Optimization (STBO), which has applications in handling optimization tasks. The fundamental inspiration of STBO is teaching the process of sewing to beginner tailors. The theory of the proposed STBO approach is described and then mathematically modeled in three phases: (i) training, (ii) imitation of the instructor’s skills, and (iii) practice. STBO performance is evaluated on fifty-two benchmark functions consisting of unimodal, high-dimensional multimodal, fixed-dimensional multimodal, and the CEC 2017 test suite. The optimization results show that STBO, with its high power of exploration and exploitation, has provided suitable solutions for benchmark functions. The performance of STBO is compared with eleven well-known metaheuristic algorithms. The simulation results show that STBO, with its high ability to balance exploration and exploitation, has provided far more competitive performance in solving benchmark functions than competitor algorithms. Finally, the implementation of STBO in solving four engineering design problems demonstrates the capability of the proposed STBO in dealing with real-world applications.

## Introduction

Optimization problems represent challenges with several possible solutions, one of which is the best choice. Accordingly, optimization is the process of achieving the best solution to the optimization problem. An optimization problem has three main parts: decision variables, objective function, and constraints^1^. Optimization aims to determine the values of the decision variables by considering the constraints so that the objective function is optimized^2^. Optimization problem-solving methods fall into two groups, deterministic and random approaches. Deterministic approaches deal well with linear, continuous, differentiable, and convex optimization problems. However, the disadvantage of these approaches is that their ability is lost in solving nonlinear, non-convex, non-differentiable, high-dimensional, NP-hard problems and discrete search spaces. These items, which have led to the inability of deterministic approaches, are among the features of real-world optimization problems. Stochastic algorithms, especially metaheuristic algorithms, have been introduced to meet this challenge. Metaheuristic algorithms can provide suitable solutions to optimization problems by using random search in problem-solving space and relying on random operators^3^. The critical thing about metaheuristic algorithms is that there is no guarantee that the solution obtained from these methods will be the best or global optimal. This fact has led researchers to develop numerous metaheuristic algorithms to achieve better solutions.

Metaheuristic algorithms are designed based on modeling ideas that exist in nature. Among the most famous metaheuristic algorithms can be mentioned Genetic Algorithm (GA)^4^, Particle Swarm Optimization (PSO)^5^, Ant Colony Optimization (ACO)^6^, and Artificial Bee Colony (ABC)^7^. GA is based on modeling the reproductive process. PSO is developed based on modeling the swarm movement of birds and fish in nature. ACO is designed based on simulating the natural behaviors of ants, and ABC is introduced based on modeling the activities of bee colonies in search of food.

Metaheuristic algorithms must have an acceptable ability for exploration and exploitation to deliver suitable optimization performance. Exploration is the concept of global search in different parts of the problem-solving space to find the main optimal area. Exploitation means a local search around candidate solutions to find better possible solutions that may be near them. In addition to having a high quality in exploration and exploitation, balancing these two indicators is the key to the success of metaheuristic algorithms^8^.

The main research question is, despite the large number of metaheuristic algorithms introduced so far, is there still a need to introduce newer methods? The answer to this question lies in the concept of the No Free Lunch (NFL) theorem^9^. According to the NFL, the excellent performance of an algorithm in solving a set of optimization problems does not guarantee the same performance of that algorithm in other optimization problems. This result is due to the random nature of metaheuristic algorithms in achieving the solution. In other words, the NFL states that it is impossible to claim that a particular algorithm is the best optimizer for dealing with all optimization issues. As a result, the NFL theorem has encouraged researchers to design new algorithms to provide more appropriate solutions closer to global optimization problems. The NFL has also motivated the authors of this study to be able to solve optimization problems more effectively by designing a new metaheuristic algorithm.

The novelty and innovation of this paper are in designing a new algorithm called Sewing Training-Based Optimization (STBO) for optimization applications. The main contributions of this article are as follows:

- A new human-based metaheuristic algorithm based on sewing training modeling is introduced.
- STBO is modeled in three phases: (i) training, (ii) imitation of the instructor's skills, and (iii) practice.
- STBO performance is tested on fifty-two benchmark functions of unimodal, high-dimensional, fixed-dimensional multimodal types and from the CEC 2017 test suite.
- STBO results are compared with the performance of eleven well-known metaheuristic algorithms.
- STBO's performance in solving real-world applications is evaluated on four engineering design issues.

The rest of the paper is organized so that a literature review is presented in the section “Literature review”. Next, the proposed algorithm is introduced and modeled in the section “Sewing Training-Based Optimization”. Simulations and analysis of their results are presented in the section “Simulation Studies and Results”. The STBO's performance in solving real-world problems is shown in the section “STBO for real-world applications.” Finally, conclusions and several study proposals are provided in the section “Conclusion and future works”.

## Literature review

Metaheuristic algorithms have been developed based on mathematical simulations of various natural phenomena, animal behaviors, biological sciences, physics concepts, game rules, human behaviors, and other evolution-based processes. Based on the source of inspiration used in the design, metaheuristic algorithms fall into five groups: swarm-based, evolutionary-based, physics-based, game-based, and human-based.

Swarm-based algorithms are derived from the mathematical modeling of natural swarming phenomena, the behavior of animals, birds, aquatic animals, insects, and other living organisms. For example, ant colonies can find an optimal path to supply the required food resources. Simulating this behavioral feature of ants forms the basis of ACO. Fireflies' feature of emitting flashing light and the light communication between them has been a source of inspiration in the design of the Firefly Algorithm (FA)^10^. Swarming activities such as foraging and hunting among animals are intelligence processes that are employed in the design of various algorithms such as PSO, ABC, Grey Wolf Optimizer (GWO)^11^, Whale Optimization Algorithm (WOA)^12^, Marine Predator Algorithm (MPA)^13^, Cat and Mouse based Optimizer (CMBO)^14^, Tunicate Swarm Algorithm (TSA)^15,16^, Reptile Search Algorithm (RSA)^17^, and Orca Predation Algorithm (OPA)^18^. Other swarm-based methods are Farmland Fertility^19^, African Vultures Optimization Algorithm (AVOA)^20^, Artificial Gorilla Troops Optimizer (GTO)^21^, Tree Seed Algorithm (TSA)^22^, Spotted Hyena Optimizer (SHO)^23^, and Pelican Optimization Algorithm (POA)^24^.

Evolutionary-based algorithms are inspired by the biological sciences, the concept of natural selection, and random operators. For example, Differential Evolution (DE)^25^ and GA are two of the most significant evolutionary algorithms developed based on the mathematization of the reproductive process, concepts of Darwin's theory of evolution, and random operators of selection, mutation, and crossover. Some other Evolutionary-based algorithms are Genetic Programming (GP)^26^, Memetic Algorithm (MA)^27^, Evolution Strategy (ES)^28^, Evolutionary Programming (EP)^29^, and Cultural Algorithm (CA)^30^.

Physics-based algorithms have been developed by simulating various laws, concepts, forces, and phenomena in physics. For example, the physical phenomenon of the water cycle has been the main idea in designing Water Cycle Algorithm (WCA)^31^. The employment of physical forces to design metaheuristic algorithms has been successful in designing algorithms such as Gravitational Search Algorithm (GSA)^32^, Spring Search Algorithm (SSA)^33^, and Momentum Search Algorithm (MSA)^34^. GSA is based on modeling the gravitational force that exists between masses at different distances from each other. SSA is inspired by the simulation of the spring tensile force and the Hook law between the weights connected by springs. MSA is developed based on the mathematization of the force of bullets' momentum that moves toward the optimal solution. Simulated Annealing (SA)^35^, Flow Regime Algorithm (FRA)^36^, Equilibrium Optimizer (EO)^37^, and Multi-Verse Optimizer (MVO)^38^ belong, e.g., among some other physics-based metaheuristic algorithms.

Game-based algorithms are formed by mathematical modeling of various game rules. For example, Volleyball Premier League (VPL) algorithm^39^ and Football Game-Based Optimization (FGBO)^40^ are game-based algorithms designed based on the simulation of club competitions in volleyball and football games, respectively. Likewise, the players' attempt in the tug-of-war game has been the main inspiration for the Tug of War Optimization (TWO)^41^ design. Likewise, the skill and strategy of the players in completing the puzzle pieces have been the idea behind the Puzzle Optimization Algorithm (POA)^42^ design.

Human-based algorithms have emerged inspired by human behaviors and interactions. This group's most widely used and well-known algorithm is Teaching–Learning-Based Optimization (TLBO). TLBO is introduced based on the mathematization of educational interactions between teachers and students^43^. The treatment process that the doctor uses to treat patients has been a central idea in the design of the Doctor and Patients Optimization (DPO)^44^. The relationships and collaboration of team members to perform teamwork and achieve the planned goal have been the source of inspiration for the Teamwork Optimization Algorithm (TOA) design^45^. Some other human-based metaheuristic algorithms are Society Civilization Algorithm (SCA)^1^, Seeker Optimization Algorithm (SOA)^46^, Imperialist Competitive Algorithm (ICA)^47^, Human-Inspired Algorithm (HIA)^48^, Social Emotional Optimization Algorithm (SEOA)^49^, Brain Storm Optimization (BSO)^50^, Anarchic Society Optimization (ASO)^51^, Human Mental Search (HMS)^52^, Gaining Sharing Knowledge based Algorithm (GSK)^53^, Coronavirus Herd Immunity Optimizer (CHIO)^54^, Ali Baba and the Forty Thieves (AFT)^55^, Human Mental Search (HMS)^52^, Multi-Leader Optimizer (MLO), Poor and Rich Optimization (PRO)^56^, Following Optimization Algorithm (FOA)^57^, and Election-Based Optimization Algorithm (EBOA)^58^.

Scientists' research in metaheuristic algorithm studies also includes improving existing algorithms^59–63^, extending hybrid algorithms by combining different algorithms to increase their efficiency^64^, developing binary versions of optimization algorithms^65–68^, and comprehensive survey studies^69–71^.

Several more recent or well-known metaheuristic algorithms published by researchers are listed in Table 1. In addition, this table specifies these algorithms' categories and sources of inspiration.

**Table 1:** A brief review of metaheuristic algorithms.

Category	Algorithm	Inspiration
Swarm-based	Particle Swarm Optimization (PSO)^5^	Searching flocks of birds and fish for food
	Ant Colony Optimization (ACO)^6^	Ant colony behavior in identifying the shortest path
	Artificial Bee Colony (ABC)^7^	Colony behavior of honey bees in holding food resources
	Firefly Algorithm (FA)^10^	Social behavior of fireflies
	Grey Wolf Optimizer (GWO)^11^	Hierarchical behavior of gray wolves during hunting
	Whale Optimization Algorithm (WOA)^12^	Social behavior of humpback whales
	Marine Predator Algorithm (MPA)^13^	The strategy of marine predators in hunting
	Cat and Mouse based Optimizer (CMBO)^14^	The process of chasing mice by cats
	Tunicate Swarm Algorithm (TSA)^15,16^	Jet propulsion and swarm intelligence of tunicate swarm during the searching for a food source
	Reptile Search Algorithm (RSA)^17^	Hunting behavior of Reptiles
	Orca Predation Algorithm (OPA)^18^	Predatory behavior of orcas
	Farmland Fertility^19^	Farmland fertility in nature
	African Vultures Optimization Algorithm (AVOA)^20^	African vultures’ lifestyle
	Artificial Gorilla Troops Optimizer (GTO)^21^	Gorilla troops' social intelligence in nature
	Tree Seed Algorithm (TSA)^22^,	Relations between trees and their seeds
	Spotted Hyena Optimizer (SHO)^23^	Social behavior of spotted hyenas
	Pelican Optimization Algorithm (POA)^24^	The strategy of pelicans when hunting prey
Evolutionary-based	Genetic Algorithm (GA)^4^	Evolutionary concepts
	Differential Evolution (DE)^25^	Darwin’s theory of evolution
	Genetic Programming (GP)^26^	Biological evolution
	Memetic Algorithm (MA)^27^	Darwinian principles and Dawkins’s notion of a meme
	Evolution Strategy (ES)^28^	Biological evolution
	Evolutionary Programming (EP)^29^	Finite state machine
	Cultural Algorithm (CA)^30^	Human cultural evolution process
Physics-based	Water Cycle Algorithm (WCA)^31^	The natural cycle of water
	Gravitational Search Algorithm (GSA)^32^	Gravitational attraction force
	Spring Search Algorithm (SSA)^33^	The tensile force of spring and Hooke's law
	Momentum Search Algorithm (MSA)^34^	The momentum of the impact of the bullets
	Simulated Annealing (SA)^35^	Metal annealing process
	Flow Regime Algorithm (FRA)^36^	Classical fluid mechanics and flow regimes
	Equilibrium Optimizer (EO)^37^	Mass balance models
	Multi-Verse Optimizer (MVO)^38^	Multi-verse theory
Game-based	Volleyball Premier League (VPL)^39^	Competition among volleyball teams during a season and coaching process during a volleyball match
	Football Game-Based Optimization (FGBO)^40^	Holding football league matches
	Tug of War Optimization (TWO)^41^	Game tug of war
	Puzzle Optimization Algorithm (POA)^42^	The effort of the players in completing the puzzle
	Ring Toss Game Based Optimizer (RTGBO)^72^	The effort of the players in throwing the ring towards the score rings
	Orientation Search Algorithm (OSA)^73^	Changing the direction of movement of players on the playground to the direction determined by the referee
	Dice Game Optimizer (DGO)^74^	Rules of the dice game
	Darts Game Optimizer (DGO)^75^	The effort of the players to earn points in the darts game
Human-based	Teaching–Learning-Based Optimization (TLBO)^43^	Teaching and learning in a classroom
	Society Civilization Algorithm (SCA)^1^	Leadership phenomena of humans
	Seeker Optimization Algorithm (SOA)^46^	The action of human randomized search
	Imperialist Competitive Algorithm (ICA)^47^	Imperialistic competition
	Human-Inspired Algorithm (HIA)^48^	People’s intelligence
	Social Emotional Optimization Algorithm (SEOA)^49^	Human social behaviors
	Brain Storm Optimization (BSO)^50^	Brainstorming process
	Anarchic Society Optimization (ASO)^51^	A social group behaving in a chaotic way to improve its situation
	Human Mental Search (HMS)^52^	Exploration strategies of the bid space in online auctions
	Gaining Sharing Knowledge based Algorithm (GSK)^53^	Acquisition and exchange of knowledge during a person’s lifespan
	Coronavirus Herd Immunity Optimizer (CHIO)^54^	Herd immunity concept to respond to COVID-19
	Ali Baba and the Forty Thieves (AFT)^55^	The tale of Ali Baba and the forty thieves
	Doctor and Patients Optimization (DPO)^44^	Interactions between doctor and patient
	Teamwork Optimization Algorithm (TOA)^45^	Teamwork of individuals in presenting their work
	Multi-Leader Optimizer (MLO)^76^	The presence of several leaders to guide the society
	Poor and Rich Optimization (PRO)^56^	Efforts of the two groups of the poor and the rich to achieve wealth and improve their economic situation
	Following Optimization Algorithm (FOA)^57^	Society people follow the successful person of the society
	Election-Based Optimization Algorithm (EBOA)^58^	The process of holding elections in society

Based on the best knowledge from the literature review, modeling the sewing training process has not been applied to designing any metaheuristic algorithm. However, sewing training by a training instructor to beginner tailors is an intelligent human activity that has the potential to simulate an optimizer. Therefore, a new human-based metaheuristic algorithm based on mathematical modeling of sewing training is designed in this paper to address this research gap. The design of this algorithm will be discussed in the next section.

### Sewing training-based optimization

This section introduces the proposed Sewing Training-Based Optimization (STBO) algorithm and presents its mathematical model.

#### Inspiration and main idea of STBO

The activity of teaching sewing skills by a training instructor to beginner tailors is an intelligent process. The first step for a beginner is to choose a training instructor. Selecting the training instructor is essential in improving a beginner's sewing skills. Next, the instructor teaches sewing techniques to the beginner tailor. The second step in this process is the beginner tailor's efforts to mimic the skills of the training instructor. The beginner tailor tries to bring his skills to the level of the instructor as much as possible. The third step in the sewing training process is practice. The beginner tailors try to improve their skills in sewing by practicing. The interactions between beginner tailors and training instructors indicate the high potential of the sewing training process to be considered for designing an optimizer. Mathematical modeling of these intelligent interactions is the fundamental inspiration in the design of STBO.

#### Mathematical model of STBO

The proposed STBO algorithm is a population-based metaheuristic algorithm whose members are beginner tailors and training instructors. Each member of the STBO population refers to a candidate solution to the problem that represents the proposed values for the decision variables. As a result, each STBO member can be mathematically modeled with a vector and the STBO population using a matrix. The STBO population is specified by a matrix representation in Eq. (1).

1 $$ X = \left[ {\begin{array}{*{20}c} {X_{1} } \\ \vdots \\ {X_{i} } \\ \vdots \\ {X_{N} } \\ \end{array} } \right]_{N \times m} = \left[ {\begin{array}{*{20}c} {x_{1,1} } & \cdots & {x_{1,j} } & \cdots & {x_{1,m} } \\ \vdots & \ddots & \vdots & {\mathinner{\mkern2mu\raise1pt\hbox{.}\mkern2mu \raise4pt\hbox{.}\mkern2mu\raise7pt\hbox{.}\mkern1mu}} & \vdots \\ {x_{i,1} } & \cdots & {x_{i,j} } & \cdots & {x_{i,m} } \\ \vdots & {\mathinner{\mkern2mu\raise1pt\hbox{.}\mkern2mu \raise4pt\hbox{.}\mkern2mu\raise7pt\hbox{.}\mkern1mu}} & \vdots & \ddots & \vdots \\ {x_{N,1} } & \cdots & {x_{N,j} } & \cdots & {x_{N,m} } \\ \end{array} } \right]_{N \times m} , $$

where $X$ is the STBO population matrix, $X_{i}$ is the *i*th STBO’s member, $N$ is the number of STBO population members, and $m$ is the number of problem variables. At the beginning of the STBO implementation, all population members are randomly initialized using Eq. (2).

2 $$ x_{i,j} = lb_{j} + r \cdot \left( {ub_{j} - lb_{j} } \right),\;i = 1,2, \ldots ,N,j = 1,2, \ldots ,m, $$

where $x_{i,j}$ is the value of the *j*th variable determined by the *i*th STBO’s member $X_{i}$, $r$ is a random number in the interval $\left[ {0,1} \right]$, $lb_{j}$ and $ub_{j}$ are the lower and upper bound of the *j*th problem variable, respectively.

Each STBO member represents a candidate solution to the given problem. Therefore, the problem's objective function can be evaluated based on the values specified by each candidate solution. Based on the placement of candidate solutions in the problem variables, the values calculated for the objective function can be modeled using a vector by Eq. (3).

3 $$ F = \left[ {\begin{array}{*{20}c} {F_{1} } \\ \vdots \\ {F_{i} } \\ \vdots \\ {F_{N} } \\ \end{array} } \right]_{N \times 1} = \left[ {\begin{array}{*{20}c} {F\left( {X_{1} } \right)} \\ \vdots \\ {F\left( {X_{i} } \right)} \\ \vdots \\ {F\left( {X_{N} } \right)} \\ \end{array} } \right]_{N \times 1} , $$

where $F$ is the objective function vector and $F_{i}$ is the objective function value for the *i*th candidate solution.

The values of the objective function are the main criterion for comparing candidate solutions with each other. The solution with the best value for the objective function is identified as the best candidate solution or the best member of the population $X_{best}$. Updating the algorithm's population in each iteration leads to finding new objective function values. Accordingly, in each iteration, the best candidate solution must be updated. The design of the algorithm guarantees that the best candidate solution at the end of each iteration is also the best candidate solution from all previous iterations.

The process of updating candidate solutions in STBO is performed in three phases: (i) training, (ii) imitation of the instructor’s skills, and (iii) practice.

##### Phase 1: Training (exploration)

The first phase of updating STBO members is based on simulating the process of selecting a training instructor and acquiring sewing skills by beginner tailors. For each STBO member as a beginner tailor, all other members with a better value for the objective function are considered training instructors for that member. The set of all candidate members as the group of possible training instructors for each STBO member $X_{i} , i = 1,2, \ldots ,N,$ is defined using the following identity

4 $$ CSI_{i} = \left\{ {X_{k} | F_{k} < F_{i} , k \in \left\{ {1,2, \ldots , N} \right\}} \right\} \cup \left\{ {X_{best} } \right\}, $$

where $CSI_{i}$ is the set of all possible candidate training instructors for the *i*th STBO member. In the case $X_{i} = X_{best}$ the only possible candidate training instructor is $X_{best}$ itself, i. e., $CSI_{i} = \left\{ {X_{best} } \right\}.$ Then, for each $i \in \left\{ {1,2, \ldots ,N} \right\}$, a member from the set $CSI_{i}$ is randomly selected as the training instructor of the *i*th member of STBO, and it is denoted as $SI_{i}$. This selected instructor $SI_{i}$ teaches the *i*th STBO member to sewing skills. Guiding members of the population under the guidance of instructors allows the STBO population to scan different areas of the search space to identify the main optimal area. This STBO update phase demonstrates the proposed approach's exploration ability in global search. At first, a new position for each population member is generated using Eq. (5) to update population members based on this phase of the STBO.

5 $$ x_{i,j}^{P1} = x_{i,j} + r_{i,j} \cdot \left( {SI_{i,j} - I_{i,j} \cdot x_{i,j} } \right) , $$

where $x_{i,j}^{P1}$ is its *d*th dimension, $F_{i}^{P1}$ is its objective function value, $I_{i,j}$ are numbers that are selected randomly from the set $\left\{ {1,2} \right\}$, and $r_{i,j}$ are random numbers from the interval $\left[ {0,1} \right]$.

Then, if this new position improves the objective function value, it replaces that population member's previous position. This update condition is modeled using Eq. (6).

6 $$ X_{i} = \left\{ {\begin{array}{*{20}l} {X_{i}^{P1} ,} \hfill & {F_{i}^{P1} < F_{i} ;} \hfill \\ {X_{i} ,} \hfill & {else,} \hfill \\ \end{array} } \right. $$

where $X_{i}^{P1}$ is the new position of the *i*th STBO member based on the first phase of STBO.

##### Phase 2: Imitation of the instructor skills (exploration)

The second phase of updating STBO members is based on simulating beginner tailors trying to mimic the skills of instructors. In the design of STBO, it is assumed that the beginner tailor tries to bring his sewing skills to the level of the instructor as much as possible. Given that each STBO member is a vector of the dimension $m$ and each component represents a decision variable thus, in this phase of STBO, it is assumed that each decision variable represents a sewing skill. Each STBO member imitates $m_{s}$ skills of the chosen instructor, $1 \le m_{s} \le m.$ This process moves the population of the algorithm to different areas in the search space, which indicates the STBO exploration ability. The set of variables that each STBO member imitates (i.e., the set of skills of the training instructor) is specified in Eq. (7).

7 $$ SD_{i} = \left\{ {d_{1} , d_{2} , \ldots ,d_{{m_{s} }} } \right\}, $$

where $SD_{i}$ is an $m_{s} -$ combination of the set $\left\{ {1,2, \ldots ,m} \right\}$, which represents the set of the indexes of decision variables (i.e., skills) identified to imitate by the *i*th member from the instructor and $m_{s} = 1 + \frac{t}{2T}m$ is the number of skills selected to mimic, $t$ is the iteration counter, and $T$ is the total number of iterations.

The new position for each STBO member is calculated based on the simulation of imitating these instructor skills, using the following identity

8 $$ x_{i,j}^{P2} = \left\{ {\begin{array}{*{20}l} {SI_{i,j} , } \hfill & {j \in SD_{i} ;} \hfill \\ {x_{i,j} ,} \hfill & {else,} \hfill \\ \end{array} } \right. $$

where $X_{i}^{P2}$ is the newly generated position for the *i*th STBO member based on the second phase of STBO, $x_{i,j}^{P2}$ is the *d*th dimension of $X_{i}^{P2} .$ This new position replaces the previous position of the corresponding member if it improves the value of the objective function

9 $$ X_{i} = \left\{ {\begin{array}{*{20}l} {X_{i}^{P2} ,} \hfill & {F_{i}^{P2} < F_{i} ;} \hfill \\ {X_{i} ,} \hfill & {else,} \hfill \\ \end{array} } \right. $$

where $F_{i}^{P2}$ is the objective function value of $X_{i}^{P2}$.

##### Phase 3: Practice (exploitation)

The third phase of updating STBO members is based on simulating beginner tailoring practices to improve sewing skills. In fact, in this phase of STBO design, a local search is performed around candidate solutions with the goal to find the best possible solutions near these candidate solutions. This phase of the STBO represents the exploitation capability of the proposed algorithm in local search. In order to mathematically model this STBO phase (with a correction to stay the all newly computed population members in the given search space), a new position around each member of the STBO is first generated using Eq. (10).

10 $$ x_{i,j}^{P3} = \left\{ {\begin{array}{*{20}l} {lb_{j} ,} \hfill & {x_{i,j}^{*} < lb_{j} ;} \hfill \\ {x_{i,j}^{*} ,} \hfill & {x_{i,j}^{*} \in \left[ {lb_{j} , ub_{j} } \right];} \hfill \\ {ub_{j} , } \hfill & {x_{i,j}^{*} > ub_{j} ,} \hfill \\ \end{array} } \right. $$

where $x_{i,j}^{*} = x_{i,j} + \left( {lb_{j} + r_{i,j} \left( {ub_{j} - lb_{j} } \right)/t} \right)$ and $r_{i,j}$ is a random number from the interval $\left[ {0,1} \right].$ Then, if the value of the objective function improves, it replaces the previous position of the STBO member according to Eq. (11).

11 $$ X_{i} = \left\{ {\begin{array}{*{20}l} {X_{i}^{P3} ,} \hfill & {F_{i}^{P3} < F_{i} ;} \hfill \\ {X_{i} ,} \hfill & {else,} \hfill \\ \end{array} } \right. $$

where $X_{i}^{P3}$ is the new generated position for the *i*th STBO member based on second phase of STBO, $x_{i,j}^{P3}$ is its *d*th dimension, and $F_{i}^{P3}$ is its objective function value.

#### Repetition process and pseudo-code of STBO

The first STBO iteration is completed after updating all candidate solutions based on the first to third phases. Then the update process is repeated until the last iteration of the algorithm, based on Eqs. (4) to (11). After the full implementation of the STBO on the given problem, the best candidate solution recorded during the algorithm iteration is introduced as the solution. Finally, STBO implementation steps are presented as pseudo-code in Algorithm 1.
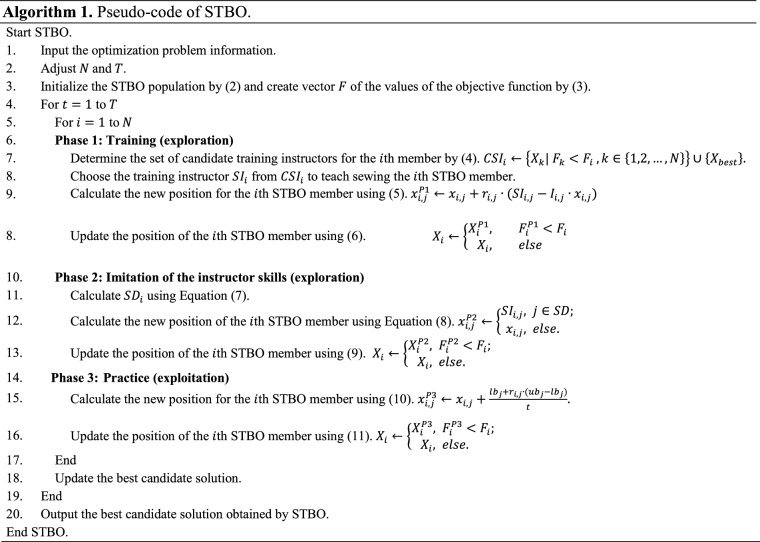


#### Computational complexity of STBO

In this subsection, the computational complexity of STBO is investigated. Since the most time-consuming step in the entire algorithm is calculating the values of the objective function, which are very complicated in most real applications, the computational complexity of STBO can be estimated based on the number of population members generated in the algorithm. STBO initialization has a computational complexity equal to $O\left(Nm\right),$ where $N$ is the number of STBO members and $m$ is the number of problem variables. In each STBO iteration, the candidate solution is updated in three phases. Thus, the computational complexity of the STBO update process is equal to $O\left(3NmT\right),$ where $T$ is the number of iterations of the algorithm. As a result, the total computational complexity of STBO is equal to $O(Nm (1+3T))$.

### Simulation Studies and Results

In this section, the ability of the proposed STBO algorithm in optimization applications and solution presentation is evaluated. In this regard, fifty-two standard benchmark functions consisting of twenty-three objective functions of unimodal, high-dimensional multimodal, fixed-dimensional multimodal types and twenty-nine benchmark functions from the CEC 2017 test suite^77^ are employed to test the STBO optimization capability^29^. The performance of DTBO is compared with the performance of eleven well-known metaheuristic algorithms GA, PSO, GSA, MPA, WOA, TLBO, RSA, MVO, GWO, AVOA, and TSA. Each of the competing metaheuristic algorithms and STBO is used in twenty independent runs, where each run contains 1000 iterations. The implementation results of metaheuristic algorithms are reported using six statistical indicators: mean, standard deviation (std), best, worst, median, and rank. The mean of rank is considered a ranking criterion of the performance of optimization algorithms for each objective function. The values of the control parameters of competitor metaheuristic algorithms are listed in Table 2.

**Table 2:** Assigned values to the control parameters of competitor algorithms.

Algorithm	Parameter	Value
AVOA	*L*_1_, *L*_2_	0.8, 0.2
	*W*	2.5
	*P*_1_, *P*_2_, *P*_3_	0.6, 0.4, 0.6
RSA	Sensitive parameter	*β* = 0.01
	Sensitive parameter	*α* = 0.1
	Evolutionary Sense (*ES*)	*ES*: randomly decreasing values between 2 and − 2
MPA	Binary vector	*U* = 0 or 1
	Random vector	*R* is a vector of uniform random numbers in [0, 1]
	Constant number	*P* = 0.5
	Fish Aggregating Devices (FADs)	*FADs* = 0.2
TSA	c1, c2, c3	Random numbers lie in the interval [0,1]
	Pmin	1
	Pmax	4
WOA	*l* is a random number in [− 1,1]	
	*r* is a random vector in [0, 1]	
	Convergence parameter (*a*)	*a*: Linear reduction from 2 to 0
GWO	Convergence parameter (*a*)	*a*: Linear reduction from 2 to 0
	Wormhole existence probability (*WEP*)	Min(*WEP*) = 0.2 and Max(*WEP*) = 1
MVO	Exploitation accuracy over the iterations (*p*)	*p* = 1
TLBO	random number	rand is a random number from interval [0,1]
	*T*_*F*_: teaching factor	*T*_*F*_ = round [(1 + *rand*)]
GSA	Alpha	20
	G_0_	100
	Rnorm	2
	Rnorm	1
PSO	Velocity limit	10% of dimension range
	Topology	Fully connected
	Inertia weight	Linear reduction from 0.9 to 0.1
	Cognitive and social constant	(*C*_1_, *C*_2_) = (2, 2)
GA	Type	Real coded
	Mutation	Gaussian (Probability = 0.05)
	Crossover	Whole arithmetic (Probability = 0.8)
	Selection	Roulette wheel (Proportionate)

#### Evaluation of unimodal benchmark functions

The results of optimization of unimodal functions F1 to F7 using STBO and competitor algorithms are reported in Table 3. The optimization results show that STBO provides the exact optimal solution for functions F1 to F6. For optimization of function F7, STBO is the best optimizer compared to competing algorithms. The simulation results show that STBO has outperformed competitor algorithms in handling the F1 to F7 unimodal functions and has been ranked first among the compared algorithms.

**Table 3:** Evaluation results on unimodal functions.

		GA	PSO	GSA	TLBO	MVO	GWO	WOA	TSA	MPA	RSA	AVOA	STBO
F_1_	Mean	33.35136	0.181599	1.09E−16	9.67E−74	0.160829	7.38E−59	1.50E−154	2.30E−47	8.66E−50	0	0	0
	Best	19.78438	3.14E−05	5.38E−17	4.45E−77	0.092366	4.54E−61	2.90E−169	4.07E−50	3.59E−52	0	0	0
	Worst	56.28509	3.274664	2.92E−16	1.07E−72	0.252881	5.46E−58	2.60E−153	1.26E−46	5.74E−49	0	0	0
	Std	8.395292	0.729657	5.21E−17	2.84E−73	0.040725	1.36E−58	5.80E−154	4.15E−47	1.43E−49	0	0	0
	Median	33.26899	0.007379	1.02E−16	2.37E−75	0.160607	1.43E−59	1.80E−161	3.45E−48	2.18E−50	0	0	0
	Rank	10	9	7	3	8	4	2	6	5	1	1	1
F_2_	Mean	3.188112	1.419726	5.33E−08	1.11E−38	0.252866	7.55E−35	7.60E−104	2.32E−28	5.72E−28	0	1.85E−267	0
	Best	1.869152	0.080876	3.98E−08	1.43E−39	0.150256	7.14E−36	7.70E−113	1.68E−30	3.30E−30	0	0	0
	Worst	4.515361	13.77933	8.14E−08	6.19E−38	0.471703	2.23E−34	5.80E−103	3.62E−27	3.98E−27	0	3.71E−266	0
	Std	0.693334	3.034488	1.02E−08	1.58E−38	0.069403	7.49E−35	1.60E−103	8.02E−28	9.79E−28	0	0	0
	Med	3.32271	0.434611	5.30E−08	4.37E−39	0.239383	3.76E−35	6.30E−107	2.23E−29	2.02E−28	0	1.61E−289	0
	Rank	11	10	8	4	9	5	3	6	7	1	2	1
F_3_	Mean	2125.752	1094.128	480.8968	2.60E−24	12.60085	9.67E−14	22,774.89	2.13E−11	8.45E−13	0	0	0
	Best	1111.139	29.63701	218.105	3.17E−28	4.866897	2.26E−19	8159.212	8.49E−20	1.16E−19	0	0	0
	Worst	2997.68	5273.106	804.0185	2.31E−23	20.88111	1.92E−12	37,690.63	2.08E−10	7.29E−12	0	0	0
	Std	495.7954	1618.416	149.9243	5.49E−24	4.434706	4.29E−13	8502.149	6.29E−11	1.75E−12	0	0	0
	Median	2238.566	428.5796	458.5233	2.42E−25	13.20446	3.96E−17	21,453.44	2.66E−14	3.20E−14	0	0	0
	Rank	9	8	7	2	6	3	10	5	4	1	1	1
F_4_	Mean	3.210794	6.507623	0.993225	1.93E−30	0.611151	2.22E−14	37.28855	0.003514	2.71E−19	0	1.29E−264	0
	Best	2.113563	3.511675	1.27E−08	1.32E−31	0.284853	6.78E−16	1.331001	1.18E−05	7.45E−20	0	6.83E−306	0
	Worst	4.712766	10.14096	4.183409	5.42E−30	1.408459	1.06E−13	80.18979	0.016565	6.16E−19	0	2.09E−263	0
	Std	0.64983	1.970793	1.230111	1.65E−30	0.293951	3.30E−14	29.04785	0.004762	1.37E−19	0	0	0
	Med	3.135406	6.130021	0.579869	1.67E−30	0.563237	7.57E−15	26.40247	0.001683	2.83E−19	0	3.21E−286	0
	Rank	9	10	8	3	7	5	11	6	4	1	2	1
F_5_	Mean	420.6	112.2916	43.52647	26.53115	308.8081	26.83281	27.0278	28.46818	23.58215	10.13576	1.667E−05	0
	Best	227.4906	30.07967	25.05162	26.03302	27.29038	25.32729	26.49484	27.13541	23.00782	1.72E−28	3.862E−07	0
	Worst	688.7775	400.1077	177.7903	28.75063	2557.854	28.5481	27.97937	29.2537	24.95884	28.99011	6.931E−05	0
	Std	122.0165	85.2441	39.12394	0.592674	622.6938	0.947923	0.328332	0.570757	0.516114	14.17161	1.81E−05	0
	Median	386.0621	84.06704	26.38317	26.35271	31.67272	26.5829	26.96864	28.6545	23.39152	5.80E−26	9.586E−06	0
	Rank	12	10	9	5	11	6	7	8	4	3	2	1
F_6_	Mean	34.0323	0.028587	1.13E−16	1.18022	0.159222	0.640991	0.119577	3.83522	2.08E−09	6.617444	4.418E−08	0
	Best	14.51884	9.98E−06	4.11E−17	0.572861	0.084059	1.14E−05	0.011797	2.823119	9.15E−10	3.624796	4.244E−09	0
	Worst	71.07024	0.324785	2.43E−16	1.754355	0.247305	1.7184	0.366934	4.774542	6.27E−09	7.498843	1.142E−07	0
	Std	15.19874	0.074692	4.95E−17	0.316997	0.046291	0.371946	0.110996	0.566697	1.13E−09	1.018988	2.515E−08	0
	Med	29.14039	0.00087	1.02E−16	1.223701	0.16068	0.748235	0.082192	3.934263	1.91E−09	7.154133	4.048E−08	0
	Rank	12	5	2	9	7	8	6	10	3	11	4	1
F_7_	Mean	0.009847	0.166943	0.067112	0.001589	0.00965	0.00083	0.001159	0.004861	0.000518	0.000103	6.378E−05	1.24E−05
	Best	0.006283	0.082498	0.019988	0.000338	0.004902	0.000262	2.80E−06	0.002251	0.000114	1.21E−05	2.674E−06	2.33E−06
	Worst	0.01905	0.289493	0.255451	0.004146	0.020283	0.002168	0.005668	0.010801	0.001654	0.000304	0.000242	3.79E−05
	Std	0.003084	0.052796	0.050227	0.000938	0.003826	0.00054	0.001539	0.002418	0.000365	8.26E−05	6.295E−05	9.87E−06
	Median	0.009485	0.159235	0.05478	0.001583	0.008695	0.000638	0.000632	0.004281	0.00041	9.22E−05	4.037E−05	8.63E−06
	Rank	10	12	11	7	9	5	6	8	4	3	2	1
Sum rank		73	64	52	33	57	36	45	49	31	21	14	7
Mean rank		10.428571	9.1428571	7.4285714	4.7142857	8.1428571	5.1428571	6.4285714	7	4.4285714	3	2	1
Total rank		12	11	9	5	10	6	7	8	4	3	2	1

#### Evaluation of high dimensional multimodal benchmark functions

The results obtained using STBO and competitor algorithms in optimizing high-dimensional multimodal functions F8 to F13 are presented in Table 4. Based on the results, STBO has provided the exact optimal solution for optimizing functions F9 and F11. Furthermore, in solving the functions F8, F10, F12, and F13, the STBO has performed better than all competitor algorithms. Analysis of the simulation results indicates the superiority of STBO over competing algorithms in handling the high-dimensional multimodal functions of F8 to F13.

**Table 4:** Evaluation results on high-dimensional multimodal functions.

		GA	PSO	GSA	TLBO	MVO	GWO	WOA	TSA	MPA	RSA	AVOA	STBO
F_8_	Mean	− 8551.34	− 6891.6	− 2463.53	− 5320.45	− 7820.6	− 6233.81	− 11,107.1	− 5989.44	− 9641.09	− 5392.18	− 12,027.8769	− 12,269.7
	Best	− 9693.59	− 8047.43	− 3421.12	− 6267.63	− 9486.17	− 7747.76	− 12,569.3	− 6812.45	− 10,493.2	− 5729.76	− 12,566.471	− 12,569.3
	Worst	− 7299.22	− 5394.56	− 1914.24	− 4471.98	− 6625.33	− 4899.26	− 8492.23	− 4918.97	− 8788.53	− 4359.71	− 10,717.161	− 11,262.6
	Std	761.0887	874.6514	363.8174	473.6453	741.5584	812.3497	1587.459	638.8189	434.3437	350.8895	499.0349695	362.218
	Median	− 8611.96	− 6843.87	− 2484.38	− 5243.46	− 7737.48	− 6303.59	− 11,928.6	− 6010.79	− 9651.7	− 5542.27	− 12,232.4668	− 12,395.1
	Rank	4	6	11	10	5	7	2	8	3	9	2	1
F_9_	Mean	58.68141	69.57591	26.01816	0	108.683	0.464705	0	170.1913	0	0	0	0
	Best	32.80754	32.85466	13.92943	0	75.69692	0	0	98.82944	0	0	0	0
	Worst	79.7012	114.4199	40.79327	0	166.2595	5.02123	0	249.7671	0	0	0	0
	Std	12.70118	20.94823	6.554449	0	24.78061	1.310176	0	44.86406	0	0	0	0
	Med	56.30895	65.84376	25.37144	0	100.1247	0	0	169.0233	0	0	0	0
	Rank	4	5	3	1	6	2	1	7	1	1	1	1
F_10_	Mean	3.659085	2.869241	8.55E−09	4.26E−15	0.940669	1.72E−14	4.09E−15	1.520055	4.26E−15	8.88E−16	8.88178E−16	8.88E−16
	Best	3.045616	0.978948	6.02E−09	8.88E−16	0.087874	1.15E−14	8.88E−16	1.51E−14	8.88E−16	8.88E−16	8.88178E−16	8.88E−16
	Worst	4.366778	4.121509	1.33E−08	4.44E−15	2.915234	2.22E−14	7.99E−15	3.500347	4.44E−15	8.88E−16	8.88178E−16	8.88E−16
	Std	0.411662	0.78912	1.91E−09	7.94E−16	0.938497	3.53E−15	2.55E−15	1.572911	7.94E−16	0	0	0
	Median	3.720395	3.025406	8.15E−09	4.44E−15	0.703059	1.51E−14	4.44E−15	1.270002	4.44E−15	8.88E−16	8.88178E−16	8.88E−16
	Rank	9	8	5	3	6	4	2	7	3	1	1	1
F_11_	Mean	1.524898	0.308563	8.934676	0	0.407293	0.00097	0.003062	0.008866	0	0	0	0
	Best	1.251305	0.012446	5.463953	0	0.259112	0	0	0	0	0	0	0
	Worst	1.791288	4.090669	17.88423	0	0.603916	0.019408	0.06124	0.017366	0	0	0	0
	Std	0.143664	0.899622	3.180256	0	0.07769	0.00434	0.013694	0.005776	0	0	0	0
	Med	1.488223	0.071877	7.923337	0	0.415251	0	0	0.00982	0	0	0	0
	Rank	7	5	8	1	6	2	3	4	1	1	1	1
F_12_	Mean	0.155349	1.391328	0.21731	0.081032	0.807735	0.038726	0.024389	6.409807	1.64E−10	1.408487	3.3717E−09	1.57E−32
	Best	0.042239	0.000558	4.30E−19	0.040371	0.001623	0.012946	0.000813	0.333781	7.55E−11	0.940108	6.677E−10	1.57E−32
	Worst	0.345347	3.204317	1.494657	0.138141	3.001919	0.105321	0.330719	13.96328	3.53E−10	1.629701	1.12808E−08	1.57E−32
	Std	0.075924	1.03366	0.392116	0.025574	0.822807	0.027369	0.072623	3.761599	8.51E−11	0.24139	2.36553E−09	2.81E−48
	Median	0.1459	1.500485	0.057647	0.0787	0.440093	0.029434	0.006351	5.533201	1.38E−10	1.51496	2.84526E−09	1.57E−32
	Rank	7	10	8	6	9	5	4	12	2	11	3	1
F_13_	Mean	2.160287	3.08976	0.015448	0.905929	0.031928	0.593907	0.227661	3.0785	0.001674	0.26	2.1597E−08	1.35E−32
	Best	0.993663	0.056865	6.03E−18	0.539664	0.010281	0.274715	0.039034	1.872547	1.24E−09	1.73E−31	2.0205E−09	1.35E−32
	Worst	4.186692	12.9562	0.254017	1.577393	0.13647	1.105615	0.458168	3.93204	0.010987	2.9	1.07149E−07	1.35E−32
	Std	0.701865	3.228647	0.057034	0.230028	0.026955	0.225443	0.143038	0.558013	0.004015	0.80616	2.57234E−08	2.81E−48
	Med	2.194029	2.146609	1.23E−17	0.886316	0.026003	0.58815	0.239423	3.052745	3.12E−09	1.46E−30	1.02427E−08	1.35E−32
	Rank	10	12	4	9	5	8	6	11	3	7	2	1
Sum rank		42	47	40	31	38	29	19	50	14	31	10	6
Mean rank		7	7.8334	6.6667	5.1666	6.3333	4.8333	3.1667	8.3333	2.3333	5.1666	1.6667	1
Total rank		9	10	8	6	7	5	4	11	3	6	2	1

#### Evaluation of fixed dimensional multimodal benchmark functions

The results of the implementation of STBO and competitor algorithms on fixed-dimensional multimodal functions F14 to F23 are released in Table 5. Compared to competitor algorithms, the optimization results show that STBO is the best optimizer in optimizing benchmark functions F14, F15, and F18. In optimizing functions F16, F17, and F19 to F23, the proposed STBO, and some competitor algorithms have a similar value in the "mean" index. However, STBO provides more efficient performance in these functions by providing better values of the "std" index. The simulation results show that STBO performs better than competitor algorithms in solving fixed-dimensional functions F14 to F23.

**Table 5:** Evaluation results on fixed-dimensional multimodal functions.

		GA	PSO	GSA	TLBO	MVO	GWO	WOA	TSA	MPA	RSA	AVOA	STBO
F_14_	Mean	0.998102	3.212919	3.564456	0.998007	0.998004	4.221422	3.88582	6.56528	0.998004	4.469522	1.14691	0.998004
	Best	0.998004	0.998004	0.998004	0.998004	0.998004	0.998004	0.998004	0.998004	0.998004	1.002309	0.998004	0.998004
	Worst	0.998721	10.76318	8.840836	0.998034	0.998004	12.67051	10.76318	12.67051	0.998004	10.76318	2.982105	0.998004
	Std	0.000213	2.88548	2.189673	6.96E−06	7.30E−12	4.652866	4.146898	4.89171	1.82E−10	3.084637	0.485651	0
	Median	0.998004	2.487068	2.806896	0.998004	0.998004	1.990054	1.495017	4.948548	0.998004	2.982105	0.998004	0.998004
	Rank	5	7	8	4	2	10	9	12	3	11	6	1
F_15_	Mean	0.01273	0.001638	0.002156	0.003375	0.007576	0.001357	0.000693	0.008509	0.000307	0.001305	0.000429	0.000307
	Best	0.000767	0.000307	0.000923	0.000309	0.000336	0.000307	0.000308	0.000308	0.000307	0.000727	0.000308	0.000307
	Worst	0.026092	0.020363	0.00352	0.020364	0.020363	0.020363	0.002252	0.020942	0.000307	0.002601	0.001223	0.000307
	Std	0.010579	0.004442	0.000483	0.007325	0.009629	0.004478	0.000523	0.010056	2.92E−19	0.000536	0.000231	2.99E−19
	Med	0.012594	0.000307	0.002076	0.00032	0.000755	0.000308	0.000475	0.000779	0.000307	0.001149	0.000322	0.000307
	Rank	12	7	8	9	10	6	4	11	2	5	3	1
F_16_	Mean	− 1.03163	− 1.03163	− 1.03163	− 1.03163	− 1.03163	− 1.03163	− 1.03163	− 1.02846	− 1.03163	− 1.02844	− 1.03163	− 1.03163
	Best	− 1.03163	− 1.03163	− 1.03163	− 1.03163	− 1.03163	− 1.03163	− 1.03163	− 1.03163	− 1.03163	− 1.03162	− 1.03163	− 1.03163
	Worst	− 1.03161	− 1.03163	− 1.03163	− 1.03162	− 1.03163	− 1.03163	− 1.03163	− 1	− 1.03163	− 1	− 1.03163	− 1.03163
	Std	4.37E−06	1.14E−16	1.25E−16	2.49E−06	4.97E−08	3.27E−09	5.93E−11	0.009735	2.10E−10	0.007256	7.20E−15	7.75E−16
	Median	− 1.03163	− 1.03163	− 1.03163	− 1.03163	− 1.03163	− 1.03163	− 1.03163	− 1.03163	− 1.03163	− 1.03067	− 1.03163	− 1.03163
	Rank	6	1	1	7	5	4	2	8	3	9	1	1
F_17_	Mean	0.524411	0.539013	0.397887	0.422207	0.397887	0.397888	0.397888	0.397906	0.397887	0.638307	0.397887	0.397887
	Best	0.397887	0.397887	0.397887	0.39789	0.397887	0.397887	0.397887	0.397888	0.397887	0.397901	0.397887	0.397887
	Worst	2.791186	2.791184	0.397887	0.882291	0.397888	0.397894	0.397891	0.397971	0.397887	5.040108	0.397887	0.397887
	Std	0.534343	0.538701	0	0.108293	6.52E−08	1.63E−06	8.40E−07	2.48E−05	2.71E−09	1.036187	0	0
	Med	0.397892	0.397887	0.397887	0.397972	0.397887	0.397888	0.397887	0.397894	0.397887	0.402551	0.397887	0.397887
	Rank	8	9	1	7	3	5	4	6	2	10	1	1
F_18_	Mean	5.729191	3	3	3.000001	3	3.000011	3.000005	15.15005	3	6.169642	3.000002	3
	Best	3.000044	3	3	3	3	3	3	3	3	3	3	3
	Worst	30.53809	3	3	3.000003	3.000001	3.000033	3.000032	84.00069	3	39.23578	3.000012	3
	Std	8.39291	2.76E−15	2.92E−15	8.96E−07	2.54E−07	9.56E−06	8.89E−06	29.67426	5.39E−14	9.865771	3.08E−06	1.81E−16
	Median	3.001628	3	3	3	3	3.000009	3.000001	3.000008	3	3.000086	3.000001	3
	Rank	10	2	3	6	5	9	8	12	4	11	7	1
F_19_	Mean	− 3.86228	− 3.86278	− 3.86278	− 3.86203	− 3.86278	− 3.86096	− 3.8602	− 3.86273	− 3.86278	− 3.80846	− 3.86278	− 3.86278
	Best	− 3.86278	− 3.86278	− 3.86278	− 3.8627	− 3.86278	− 3.86278	− 3.86278	− 3.86278	− 3.86278	− 3.85487	− 3.86278	− 3.86278
	Worst	− 3.85745	− 3.86278	− 3.86278	− 3.8548	− 3.86278	− 3.8549	− 3.85378	− 3.86256	− 3.86278	− 3.68429	− 3.86278	− 3.86278
	Std	0.001431	2.09E−15	1.87E−15	0.001716	7.41E−08	0.002948	0.002953	5.09E−05	2.28E−15	0.04789	3.22E−13	2.78E−15
	Med	− 3.86277	− 3.86278	− 3.86278	− 3.86249	− 3.86278	− 3.86246	− 3.86139	− 3.86275	− 3.86278	− 3.82667	− 3.86278	− 3.86278
	Rank	5	1	1	6	3	7	8	4	1	9	2	1
F_20_	Mean	− 3.19552	− 3.29822	− 3.322	− 3.23822	− 3.2446	− 3.23965	− 3.2753	− 3.23237	− 3.322	− 2.63831	− 3.28617	− 3.322
	Best	− 3.3214	− 3.322	− 3.322	− 3.31043	− 3.32199	− 3.32199	− 3.32198	− 3.32137	− 3.322	− 3.15625	− 3.322	− 3.322
	Worst	− 2.99692	− 3.2031	− 3.322	− 3.08169	− 3.20259	− 3.02064	− 3.10782	− 2.84	− 3.322	− 1.30322	− 3.19994	− 3.322
	Std	0.093531	0.048793	3.95E−16	0.065246	0.058264	0.095719	0.074262	0.146905	4.20E−16	0.417228	0.056151	2.49E−16
	Median	− 3.18946	− 3.322	− 3.322	− 3.1998	− 3.20302	− 3.26252	− 3.32127	− 3.31998	− 3.322	− 2.73165	− 3.322	− 3.322
	Rank	9	2	1	7	5	6	4	8	1	10	3	1
F_21_	Mean	− 5.89083	− 5.77879	− 6.17737	− 5.84595	− 8.51163	− 9.64743	− 8.36636	− 6.52198	− 10.1532	− 5.0552	− 10.1532	− 10.1532
	Best	− 9.0381	− 10.1532	− 10.1532	− 9.44872	− 10.1532	− 10.1531	− 10.1529	− 10.138	− 10.1532	− 5.0552	− 10.1532	− 10.1532
	Worst	− 2.34247	− 2.63047	− 2.63047	− 3.80037	− 2.63047	− 5.10034	− 5.05374	− 2.60298	− 10.1532	− 5.0552	− 10.1532	− 10.1532
	Std	2.512564	3.703566	3.699126	1.769566	2.623726	1.55506	2.493026	3.337297	1.95E−15	2.48E−07	6.57E−15	3.65E−15
	Med	− 6.83679	− 2.68286	− 3.51696	− 5.02319	− 10.1531	− 10.1527	− 10.1469	− 5.04462	− 10.1532	− 5.0552	− 10.1532	− 10.1532
	Rank	8	10	7	9	4	3	5	6	1	11	2	1
F_22_	Mean	− 7.21825	− 6.31807	− 10.4029	− 8.09591	− 9.6056	− 10.4024	− 8.0395	− 7.53629	− 10.4029	− 5.08767	− 10.4029	− 10.4029
	Best	− 10.1952	− 10.4029	− 10.4029	− 9.92173	− 10.4029	− 10.4028	− 10.4028	− 10.3998	− 10.4029	− 5.08767	− 10.4029	− 10.4029
	Worst	− 2.62184	− 1.83759	− 10.4029	− 4.21215	− 5.08765	− 10.4018	− 2.76572	− 1.82822	− 10.4029	− 5.08767	− 10.4029	− 10.4029
	Std	2.472441	3.837031	2.97E−15	1.699295	1.947221	0.00028	3.034341	3.483042	3.65E−15	5.80E−07	2.41E−14	2.88E−15
	Median	− 7.89012	− 4.40599	− 10.4029	− 8.81648	− 10.4029	− 10.4025	− 10.3974	− 10.1566	− 10.4029	− 5.08767	− 10.4029	− 10.4029
	Rank	9	10	2	6	5	4	7	8	1	11	3	1
F_23_	Mean	− 5.78525	− 5.62285	− 10.5364	− 8.30576	− 9.99556	− 9.72457	− 8.77684	− 5.46265	− 10.5364	− 5.12847	− 10.5364	− 10.5364
	Best	− 10.417	− 10.5364	− 10.5364	− 9.98248	− 10.5364	− 10.5364	− 10.5364	− 10.4691	− 10.5364	− 5.12848	− 10.5364	− 10.5364
	Worst	− 2.38428	− 2.42173	− 10.5364	− 4.06348	− 5.12846	− 2.42172	− 2.42169	− 1.67573	− 10.5364	− 5.12847	− 10.5364	− 10.5364
	Std	2.966829	3.755817	1.73E−15	1.491856	1.664511	2.497522	3.196353	3.753624	2.51E−15	1.29E−06	7.08E−15	6.93E−16
	Med	− 6.05259	− 3.35328	− 10.5364	− 8.76487	− 10.5364	− 10.536	− 10.5338	− 2.84687	− 10.5364	− 5.12847	− 10.5364	− 10.5364
	Rank	8	9	1	7	4	5	6	10	3	11	2	1
Sum rank		80	58	33	68	46	59	57	85	21	98	30	10
Mean rank		8	5.8	3.3	6.8	4.6	5.9	5.7	8.5	2.1	9.8	3	1
Total rank		10	7	4	9	5	8	6	11	2	12	3	1

**Figure 1:**
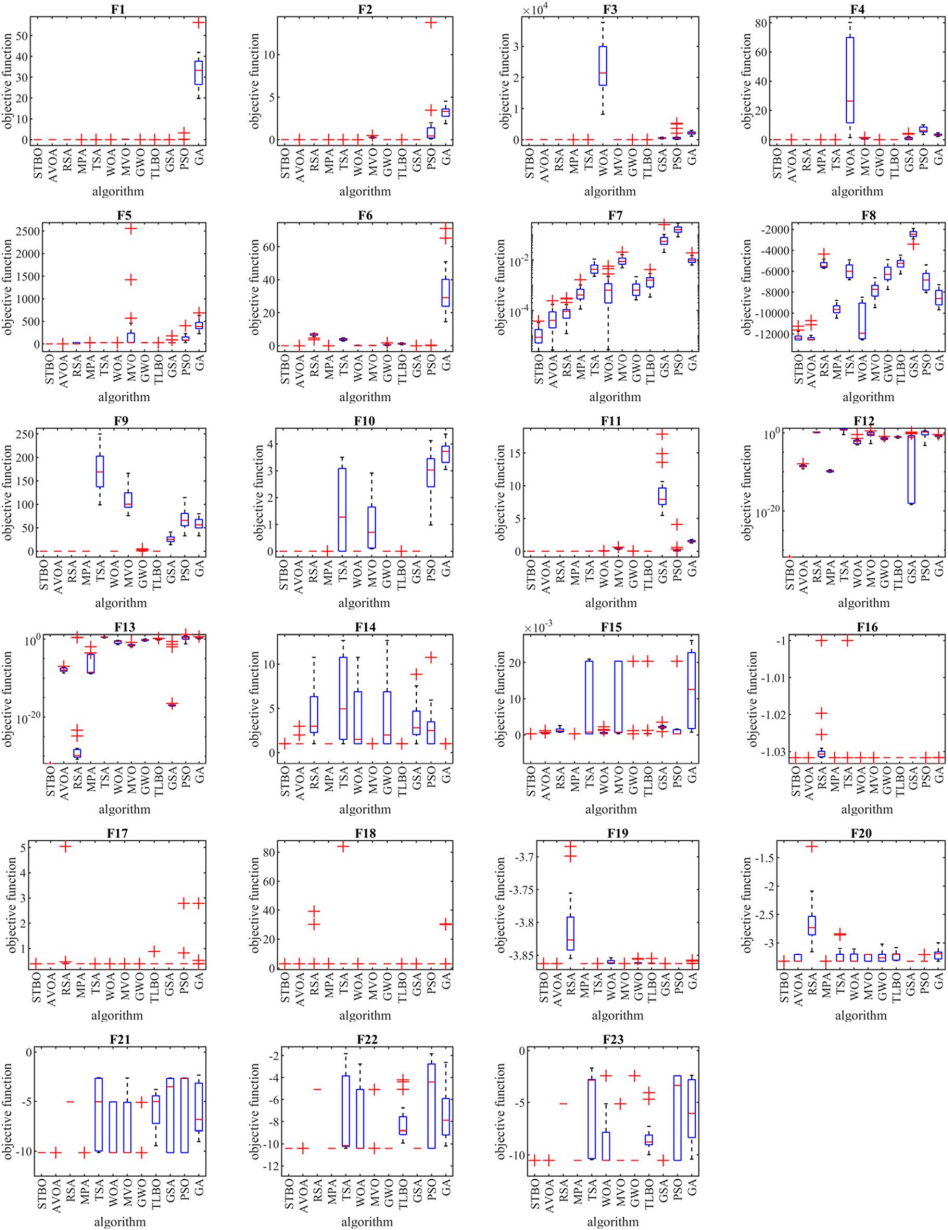
Boxplot of performance of STBO and competitor algorithms in solving F1 to F23.

The performance of STBO and competitor algorithms in optimizing F1 to F23 functions is presented as a boxplot in Fig. 1. Intuitive analysis of these boxplots shows that the proposed STBO approach has provided superior and more effective performance compared to competing algorithms by providing better results in statistical indicators in most of the benchmark functions.

### Statistical analysis

In this subsection, statistical analysis is presented to further evaluate the performance of the STBO compared to competitor algorithms. Wilcoxon sum rank test^78^ has been employed to determine whether there is a statistically significant difference between the results obtained from STBO and competing algorithms. In the Wilcoxon sum rank test, the p-value index determines the significant difference between the two data samples. The results of the Wilcoxon sum rank test on the performance of STBO and competitor algorithms are reported in Table 6. Based on these results, in cases where the $p$-value is calculated as less than 0.05, STBO has a statistically significant superiority over the competitor algorithm.

**Table 6:** Wilcoxon sum rank test results.

Compared Algorithms	Test function type		
	Unimodal	High-multimodal	Fixed-multimodal
STBO vs. AVOA	1.01E−24	1.96E−21	0.000145
STBO vs. RSA	1.01E−24	1.97E−21	0.001816
STBO vs. MPA	1.01E−24	1.97E−21	3.29E−11
STBO vs. TSA	1.01E−24	1.97E−21	0.000299
STBO vs. WOA	1.01E−24	1.04E−14	7.98E−21
STBO vs. MVO	1.01E−24	1.97E−21	4.09E−13
STBO vs. GWO	1.01E−24	7.8E−16	5.01E−07
STBO vs. TLBO	2.44E−24	9.08E−09	0.358845
STBO vs. GSA	1.01E−24	1.31E−20	1.44E−34
STBO vs. PSO	1.01E−24	1.04E−14	6.4E−10
STBO vs. GA	3.64E−11	1.63E−11	1.78E−12

### Convergence analysis

**Figure 2:**
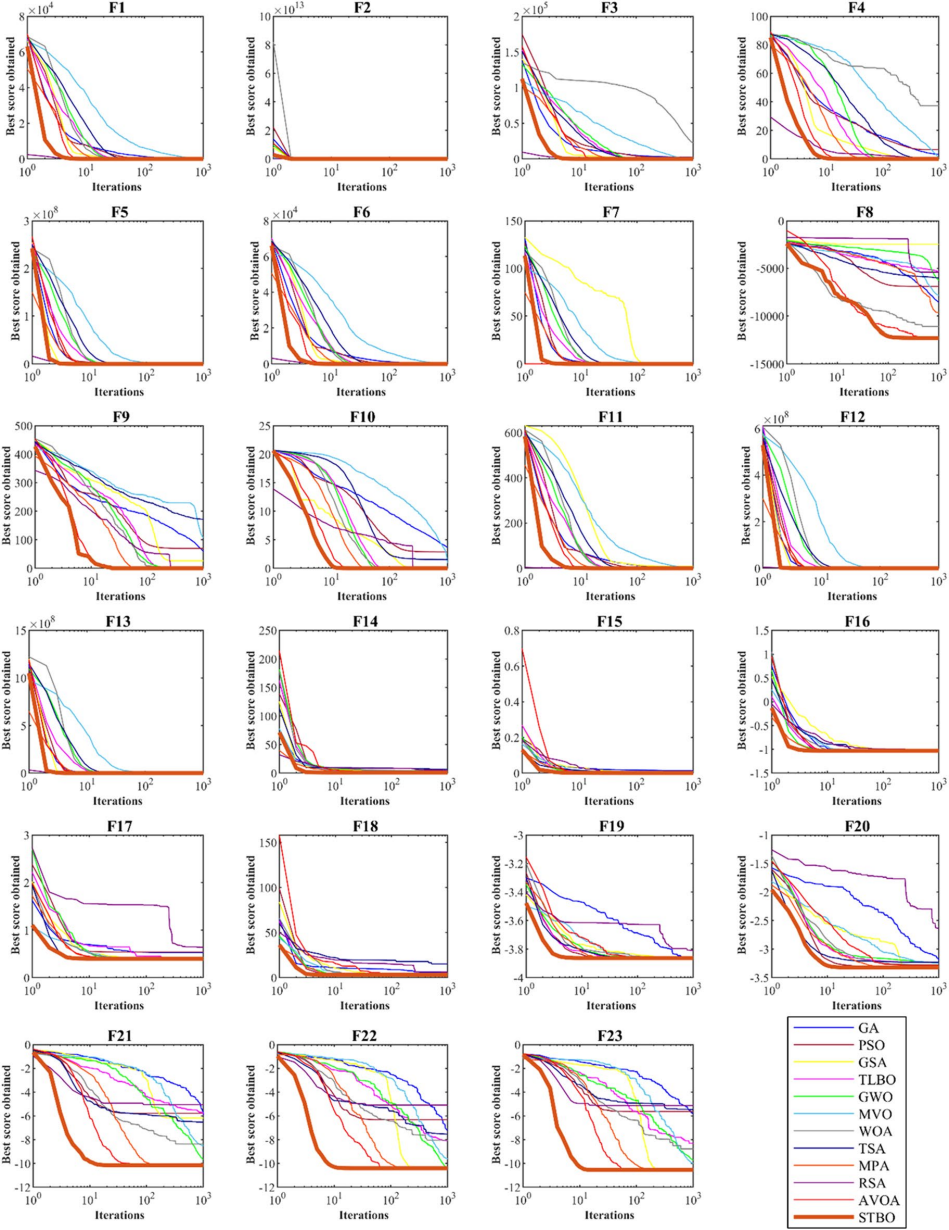
Convergence curves of STBO and competitor algorithms in solving F1 to F23.

In this subsection, the convergence analysis of the proposed STBO is presented in comparison with competitor algorithms. The convergence curves of STBO and competitor algorithms during the optimization of F1 to F23 functions are drawn in Fig. 2. In the optimization of unimodal functions F1 to F7, which lack local optima, it can be seen that STBO has converged towards better solutions with its high ability in local search and exploitation after identifying the position of the optimal solution. Especially in solving functions F1 to F6, STBO has converged to the global optimal of these functions.

In the optimization of high-dimensional multimodal functions F8 to F13, which have a large number of local optima, it can be seen that STBO with high capability in global search and exploration has been able to identify the optimal global position well without getting stuck in local areas. With increasing iterations of the algorithm, it can be seen that STBO has converged towards better solutions. Especially in optimizing F9 and F11 functions, the proposed approach, with high ability in exploration and exploitation, has converged to the global optima. In the optimization of fixed-dimension multimodal functions F14 to F23, which have a smaller number of local optima (in comparison to F8 to F13 functions), it can be seen that STBO with high ability in balancing exploration and exploitation has provided a good performance in handling these functions. STBO first identified the main optimal area in solving these functions by providing an optimal global search. Then, by increasing the number of iterations of the algorithm, using local search, it converged towards suitable solutions. Convergence analysis shows that the proposed STBO approach, with its high ability to explore and exploit and balance during algorithm iterations, has better performance in handling functions F1 to F23 compared to competitor algorithms.

### Scalability analysis

In this subsection, scalability analysis is presented to evaluate the proposed STBO approach and competitor algorithms in solving optimization problems under changes in the dimensions of the problem. In this analysis, the proposed STBO and each competing algorithm are used in optimizing the functions F1 to F13 for different dimensions $m$ equal to 50, 100, 250, and 500. The results of the scalability analysis are reported in Table 7. These found simulation results show that the efficiency of the STBO's performance does not decrease much with the increase in the dimensions of the problem. Furthermore, the scalability analysis reveals that the performance of the proposed STBO is least affected by the increase in the dimensionality of search space in comparison to competitor algorithms. This superiority is due to the proposed STBO approach's better ability to balance exploitation and exploration during the search process than competing algorithms.

**Table 7:** The results of the scalability analysis of STBO.

	$m$	Index	GA	PSO	GSA	TLBO	MVO	GWO	WOA	TSA	MPA	RSA	AVOA	STBO
F_1_	50	Mean	285.0043	34.26946	6.91E−16	5.57E−68	1.171019	8.13E−44	1.80E−151	9.37E−36	1.87E−46	0	0	0
		Std	71.44383	53.81518	4.58E−16	7.14E−68	0.247724	1.43E−43	5.50E−151	2.08E−35	4.78E−46	0	0	0
	100	Mean	2782.496	2511.523	673.4191	8.95E−62	18.86453	2.57E−29	3.10E−150	2.63E−25	3.16E−43	0	0	0
		Std	322.4319	1007.265	358.3897	1.83E−61	2.305294	2.84E−29	9.10E−150	3.68E−25	5.78E−43	0	0	0
	250	Mean	21,206.3	39,344.94	13,078.49	3.85E−58	798.4678	5.56E−18	2.00E−148	1.69E−15	1.31E−40	0	0	0
		Std	2180.106	8289.222	1581.329	6.19E−58	80.70753	3.34E−18	8.80E−148	1.96E−15	2.05E−40	0	0	0
	500	Mean	69,817.39	165,845	40,569.3	6.55E−56	12,935.45	1.39E−12	1.80E−145	1.00E−10	4.64E−39	0	0	0
		Std	3479.445	12,186.53	2491.612	1.55E−55	846.7481	6.09E−13	5.50E−145	1.08E−10	4.39E−39	0	0	0
F_2_	50	Mean	12.2036	8.359858	0.152633	3.04E−35	35.12252	4.52E−26	2.10E−104	2.23E−22	2.14E−26	0	1.10E−202	0
		Std	1.384519	4.660024	0.465052	3.51E−35	69.37835	3.78E−26	4.00E−104	6.44E−22	2.59E−26	0	0	0
	100	Mean	46.20699	123.1423	6.597008	2.04E−32	5.90E+15	4.70E−18	1.10E−101	1.53E−16	5.52E−25	0	1.50E−208	0
		Std	4.244573	88.20233	2.718062	1.38E−32	2.49E+16	2.32E−18	3.00E−101	1.72E−16	9.87E−25	0	0	0
	250	Mean	190.8026	544.4599	77.08794	4.03E−30	6.06E+85	3.31E−11	1.07E−98	2.21E−11	1.03E−23	0	7.90E−211	0
		Std	9.96777	20.69241	9.352065	2.34E−30	2.63E+86	1.12E−11	4.63E−98	1.84E−11	2.51E−23	0	0	0
	500	Mean	485.2498	1112.286	3.50E+268	3.60E−29	3.10E+211	5.96E−08	1.30E−99	2.02E−08	6.87E−21	0	3.30E−206	0
		Std	17.08654	42.27506	65,535	2.37E−29	65,535	1.15E−08	5.90E−99	2.31E−08	3.02E−20	0	0	0
F_3_	50	Mean	5048.588	6169.914	1821.115	1.29E−19	650.7283	6.38E−06	124,414.6	0.072352	2.50E−07	0	0	0
		Std	1078.129	4769.136	448.1044	4.71E−19	154.0739	2.16E−05	29,498.35	0.125204	6.11E−07	0	0	0
	100	Mean	18,325.05	50,954.41	7927.23	3.10E−15	29,633.47	11.04603	853,493.7	2220.555	0.004078	0	1.60E−280	0
		Std	3075.166	21,471.28	1361.341	5.06E−15	4515.672	19.81332	168,872.8	1432.756	0.011144	0	0	0
	250	Mean	136,756.2	336,528	57,829.84	3.66E−10	322,673.6	9257.031	6,878,229	206,030.5	107.5336	0	3.20E−241	0
		Std	23,655.14	75,834.61	8985.585	1.56E−09	21,061.8	5349.661	1,098,416	53,625.46	202.0424	0	0	0
	500	Mean	668,892.5	1,223,667	275,481.2	1.47E−08	1,317,906	116,541.8	28,062,441	1,035,782	1525.209	0	1.10E−192	0
		Std	106,978.1	359,536.2	68,207.09	5.34E−08	103,418.5	59,137.88	9,301,240	165,807.8	1673.877	0	0	0
F_4_	50	Mean	5.963966	19.53837	8.777958	1.06E−27	4.066982	1.82E−09	66.50783	2.731684	1.24E−17	0	6.70E−196	0
		Std	0.978908	3.071924	1.928206	7.94E−28	1.385832	2.88E−09	27.76152	2.912462	8.45E−18	0	0	0
	100	Mean	13.80378	37.51555	16.06594	1.35E−25	41.92272	0.003378	77.59964	34.24477	4.35E−16	0	9.20E−193	0
		Std	1.359287	3.83295	1.503749	1.33E−25	6.774704	0.003706	23.46463	12.32771	2.17E−16	0	0	0
	250	Mean	26.5063	53.88201	22.08527	6.02E−24	80.66538	21.2961	77.16777	96.01481	3.04E−14	0	1.80E−204	0
		Std	1.60905	5.368787	1.848865	1.18E−23	3.341441	7.816487	22.70411	3.384094	2.07E−14	0	0	0
	500	Mean	36.97724	67.43914	26.76013	3.20E−23	92.43218	57.82163	78.34497	99.24345	5.46E−13	0	1.80E−201	0
		Std	1.911597	4.690484	1.645312	2.84E−23	1.646392	6.138673	20.24963	0.22398	8.70E−13	0	0	0
F_5_	50	Mean	2713.719	1085.306	131.5463	47.42296	603.6723	47.40183	47.43972	48.32421	44.03808	46.49249	0.000162	0
		Std	1305.112	1169.986	73.26552	0.9332	920.3005	0.79805	0.54216	0.656619	0.590632	10.94366	0.000166	0
	100	Mean	125,110.4	287,904.9	17,204.66	97.81196	1237.219	97.63697	97.80163	98.29339	95.135	98.96354	0.00046	0
		Std	43,089.33	264,617.7	10,886.6	0.677834	844.555	0.636675	0.37934	0.488629	0.843997	0.066664	0.000509	0
	250	Mean	4,083,468	21,300,786	1,044,548	248.0782	35,979.2	247.8359	247.1009	247.9578	245.7841	248.9852	0.007509	0
		Std	1,041,684	5,177,053	294,478.5	0.299102	14,532.75	0.42663	0.319189	0.561326	0.706112	0.015649	0.018946	0
	500	Mean	21,818,155	1.66E+08	5,695,001	498.0625	3,316,483	497.6689	495.7569	498.2494	495.6662	498.9886	0.005212	0
		Std	2,517,887	25,976,011	825,086.1	0.269248	471,390.8	0.173736	0.442375	0.48665	0.528731	0.003021	0.006083	0
F_6_	50	Mean	305.615	42.04067	0.003316	3.660992	1.073896	2.243901	0.378736	6.441274	0.003395	11.46592	4.58E−05	0
		Std	77.1288	53.90269	0.014831	0.597425	0.212681	0.481915	0.230127	0.957914	0.015141	1.656754	2.24E−05	0
	100	Mean	2716.183	3158.425	649.5424	12.34831	18.44157	9.286886	1.937719	13.80242	0.815919	24.70658	0.014634	0
		Std	493.7557	2883.38	352.0708	0.75096	2.245434	0.787782	0.928162	1.00861	0.362987	0.166974	0.055799	0
	250	Mean	21,479.14	40,123.4	12,867.19	44.38734	782.2298	38.47129	8.908795	41.33491	13.65313	62.19509	0.101121	0
		Std	1771.678	6381.723	1821.69	1.271032	65.41419	1.275285	2.236528	1.601417	1.08496	0.120169	0.183189	0
	500	Mean	68,512.54	166,510.4	40,708.14	102.2647	13,141.71	92.61489	18.32255	95.38288	52.989	124.7206	0.11678	0
		Std	4438.758	14,455.79	3093.243	1.840089	725.7806	1.57135	5.184106	1.850498	1.997287	0.06375	0.234366	0
F_7_	50	Mean	0.029469	0.563845	0.192132	0.002251	0.039289	0.001623	0.003268	0.009127	0.000786	8.80E−05	0.00012	1.21E−05
		Std	0.006066	0.245682	0.070887	0.001521	0.012198	0.00081	0.004275	0.004542	0.000395	9.50E−05	0.000122	1.39E−05
	100	Mean	0.277241	6.359927	2.081773	0.002365	0.197949	0.002509	0.001804	0.022533	0.00069	4.95E−05	0.000128	1.04E−05
		Std	0.109251	6.051414	0.959512	0.001368	0.028032	0.001204	0.001604	0.008074	0.000271	7.53E−05	0.000109	1.01E−05
	250	Mean	14.51263	127.255	69.68376	0.003042	2.268139	0.00645	0.003257	0.093088	0.000885	8.38E−05	0.000157	1.36E−05
		Std	2.095997	48.34713	15.68223	0.001392	0.343546	0.00241	0.003305	0.035296	0.000421	7.11E−05	0.000179	1.18E−05
	500	Mean	152.9307	1238.266	685.4945	0.003431	41.51178	0.010719	0.002206	0.535833	0.000924	7.47E−05	0.000171	1.67E−05
		Std	26.25327	198.34	99.68845	0.002319	6.351142	0.003335	0.002982	0.122946	0.000412	7.07E−05	0.00015	1.53E−05
F_8_	50	Mean	− 12,272.8	− 10,802.7	− 3403.91	− 7204.86	− 12,793.7	− 9338.85	− 19,090.3	− 8745.76	− 14,938.1	− 9022.4	− 20,353.6	− 20,413
		Std	1103.911	1130.198	507.9481	852.2193	1008.764	1287.406	2667.391	807.3113	715.9989	202.8098	524.94	1176.142
	100	Mean	− 18,876.3	− 18,284.3	− 4539.53	− 9438.36	− 24,721	− 16,403.4	− 38,730.4	− 14,103.8	− 28,018.7	− 17,227.2	− 40,235	− 41,150
		Std	1754.896	2043.847	746.9655	961.5354	1632.216	2974.611	4599.436	889.5166	861.137	1182.774	1140.119	1847.596
	250	Mean	− 31,100.3	− 36,587.6	− 7604.78	− 15,364.1	− 55,415.3	− 35,384.6	− 87,802.5	− 23,017.4	− 57,688.4	− 37,088.8	− 96,183.8	− 100,730
		Std	3266.845	2315.238	1353.598	1984.653	2685.69	2404.494	14,060.16	1501.685	1813.612	2064.086	6413.815	4650.807
	500	Mean	− 45,608.7	− 55,847.5	− 10,442.3	− 22,573	− 96,126.1	− 61,887.1	− 188,510	− 32,328.1	− 96,995.4	− 66,008.1	− 176,969	− 201,718
		Std	3441.277	3362.234	2117.996	2518.76	2539.443	5243.808	26,903.39	1972.414	2350.496	4893.541	14,449.97	7420.386
F_9_	50	Mean	187.09	116.6431	54.67295	0	242.2961	0.49703	0	359.0799	0	0	0	0
		Std	25.47509	30.12197	9.988632	0	49.28747	1.530593	0	59.85311	0	0	0	0
	100	Mean	591.5714	340.1137	133.9363	0	572.9428	0.189209	0	897.1359	0	0	0	0
		Std	39.8328	40.68969	18.74462	0	96.95193	0.84617	0	115.5391	0	0	0	0
	250	Mean	2086.163	1457.558	693.863	0	2156.602	1.952586	2.27E−14	2711.363	0	0	0	0
		Std	64.22998	85.31377	76.61462	0	112.0258	3.110418	1.02E−13	271.96	0	0	0	0
	500	Mean	4646.933	3828.066	2231.817	0	5422.097	3.544948	0	5356.486	0	0	0	0
		Std	112.9087	150.4184	112.0155	0	163.6803	4.402665	0	486.9464	0	0	0	0
F_10_	50	Mean	5.389597	5.507252	0.286075	4.44E−15	1.754842	3.27E−14	3.91E−15	1.013365	4.44E−15	8.88E−16	8.88E−16	8.88E−16
		Std	0.379123	0.949512	0.449919	0	0.479574	3.15E−15	2.09E−15	1.424865	0	0	0	0
	100	Mean	7.96934	10.84341	3.155674	4.44E−15	5.028315	1.11E−13	4.62E−15	7.55E−10	4.44E−15	8.88E−16	8.88E−16	8.88E−16
		Std	0.3831	1.36695	0.870762	0	4.973398	6.71E−15	2.15E−15	3.37E−09	0	0	0	0
	250	Mean	10.74289	15.85386	7.168624	4.44E−15	18.97748	1.40E−10	4.62E−15	4.47E−09	4.44E−15	8.88E−16	8.88E−16	8.88E−16
		Std	0.334866	0.676188	0.371374	0	4.076866	5.24E−11	2.44E−15	3.19E−09	0	0	0	0
	500	Mean	12.25589	17.35226	9.145829	4.44E−15	20.70852	5.21E−08	3.38E−15	5.11E−07	4.44E−15	8.88E−16	8.88E−16	8.88E−16
		Std	0.174913	0.215727	0.303231	0	0.070891	1.22E−08	2.33E−15	3.81E−07	0	0	0	0
F_11_	50	Mean	4.052498	1.457349	30.33821	0	0.761331	0.000976	0	0.00581	0	0	0	0
		Std	0.803584	0.648617	7.427283	0	0.061095	0.004365	0	0.007483	0	0	0	0
	100	Mean	26.35897	21.79932	97.63246	0	1.169893	0.001514	0	0.004743	0	0	0	0
		Std	4.094358	9.647885	10.20711	0	0.024039	0.004755	0	0.007544	0	0	0	0
	250	Mean	193.6629	353.5776	1104.15	0	7.894967	0.001375	5.55E−18	0.012257	0	0	0	0
		Std	12.63072	43.8592	47.3678	0	0.713036	0.006151	2.48E−17	0.017872	0	0	0	0
	500	Mean	627.7852	1540.037	4647.865	0	121.6026	0.001047	0	0.010433	0	0	0	0
		Std	32.5443	104.1384	113.7393	0	9.110349	0.004683	0	0.02146	0	0	0	0
F_12_	50	Mean	1.794942	7.930916	1.742268	0.147094	2.719225	0.082447	0.013583	6.888216	8.49E−09	1.254882	5.36E−07	9.42E−33
		Std	0.711264	3.528295	0.894647	0.036661	0.841177	0.031664	0.014297	4.282754	1.17E−08	0.229805	3.80E−07	2.81E−48
	100	Mean	8.188648	32.71512	5.133908	0.36174	8.276208	0.24133	0.021398	10.24539	0.009444	1.258981	0.000198	4.71E−33
		Std	1.640979	24.67439	1.363192	0.040864	1.843543	0.04763	0.022961	4.540495	0.0039	0.103201	0.000879	1.40E−48
	250	Mean	47,652.44	4,716,917	21.88649	0.633515	42.14883	0.542416	0.027522	65.1672	0.072691	1.225052	9.41E−05	1.88E−33
		Std	65,626.15	2,935,210	6.096715	0.040427	7.225588	0.04906	0.010868	115.8512	0.011464	0.007345	0.000284	3.51E−49
	500	Mean	2,535,944	90,916,183	884.6464	0.824569	116,501.9	0.747753	0.039326	46,754	0.203489	1.20229	9.54E−05	9.42E−34
		Std	1,052,760	25,963,807	986.5159	0.016698	67,940.08	0.033203	0.018023	54,125.95	0.019065	0.002856	0.000205	1.76E−49
F_13_	50	Mean	12.08077	39.25416	14.53883	3.017419	0.184215	1.774649	0.585467	5.23522	0.083563	1.693285	1.48E−07	1.35E−32
		Std	3.219958	8.195503	11.08645	0.444856	0.054379	0.335202	0.263984	0.716397	0.068924	1.979402	2.09E−07	2.81E−48
	100	Mean	199.7021	29,222.48	125.1532	8.164988	80.09712	6.324213	1.986033	12.01446	6.040318	9.890124	2.54E−07	1.35E−32
		Std	225.0718	43,015.76	44.23044	0.381571	34.61998	0.424363	0.960595	1.093216	2.587355	0.03041	2.16E−07	2.81E−48
	250	Mean	1,881,794	29,219,357	98,379.96	24.24311	546.7537	21.06385	5.335894	137.2253	22.9728	24.90092	0.005026	1.35E−32
		Std	1,046,952	8,331,513	103,971.4	0.375005	71.90737	0.419527	1.264721	102.9533	0.398369	0.029412	0.022476	2.81E−48
	500	Mean	24,409,101	4.10E+08	1,078,726	49.81733	2,251,206	45.92283	11.08914	3764.666	47.63375	49.89766	0.018481	1.35E−32
		Std	5,989,248	1.06E+08	392,617.9	0.067117	781,787.8	0.540198	3.486242	3208.632	0.475035	0.011192	0.046167	2.81E−48

### Evaluation of the CEC 2017 test suite benchmark functions

**Figure 3:**
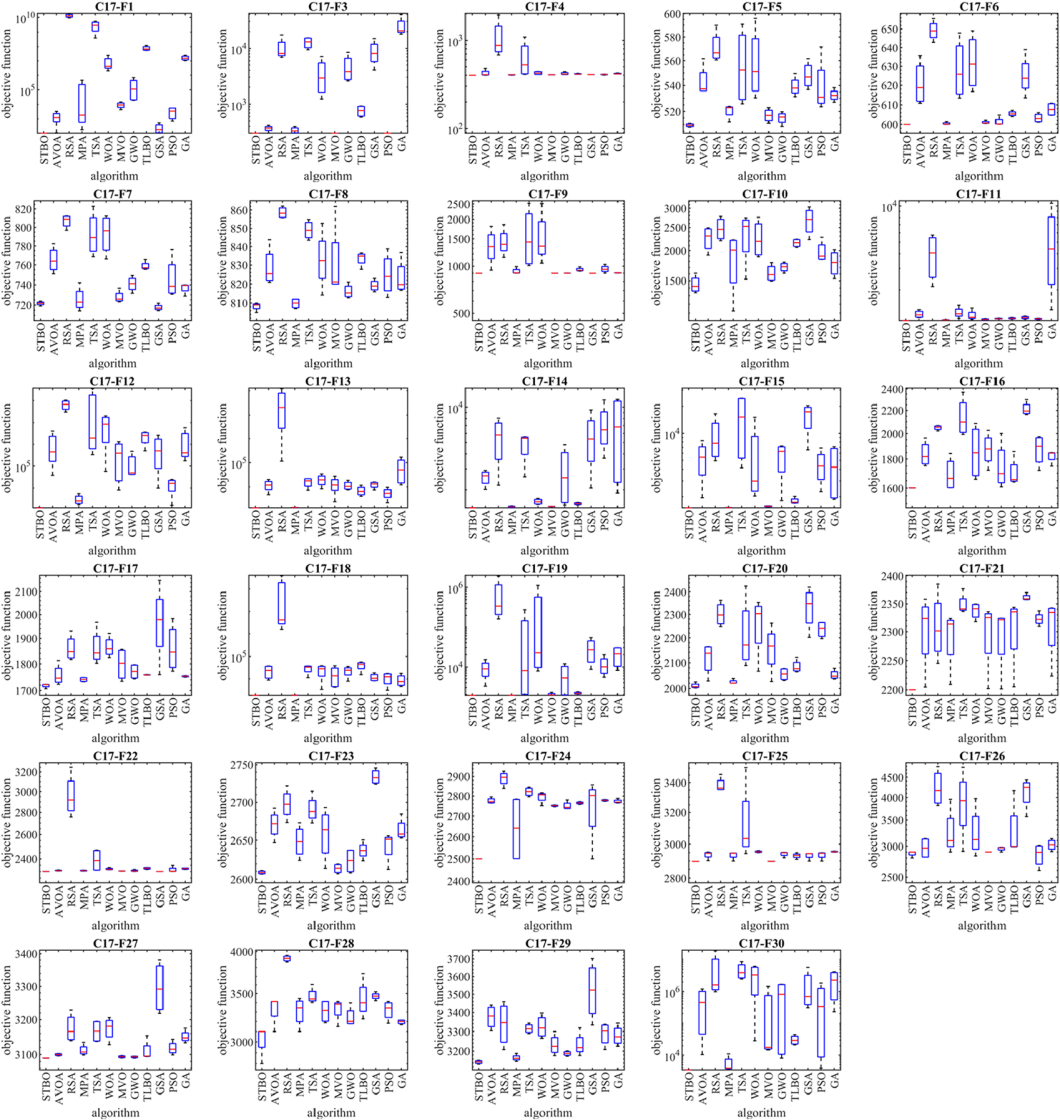
Boxplot of performance of STBO and competitor algorithms in solving the CEC 2017 test suite.

In this subsection, the performance of STBO in solving complex optimization problems of the CEC 2017 test suite is evaluated. This test suite has thirty standard benchmark functions consisting of three unimodal functions, C17-F1 to C17-F3, seven multimodal functions, C17-F4 to C17-F10, ten hybrid functions, C17-F11 to C17-F20, and ten composition functions C17- F21 to C17-F30. The C17-F2 function has been removed from this test suite due to unstable behavior. Full descriptions and details of the CEC 2017 test suite are available in the report^78^. The optimization results of the CEC 2017 test suite using the proposed STBO approach and competitor algorithms are reported in Table 8. Based on the optimization results, STBO is the first best optimizer in solving functions C17-F1, C17-F4 to C17-F6, C17-F8, C17-F10 to C17-F21, C17-F23 to C17-F25, and C17-F27 to C17-F30. The analysis of the simulation results found shows that the proposed STBO approach gives better results for most of the CEC 2017 test set features. It can be concluded that it performs better in solving this feature test set than the competing algorithms. Also, the results obtained from the Wilcoxon sum rank test show that the superiority of STBO against competitor algorithms in handling the CEC 2017 test suite is significant from a statistical point of view. The performance of STBO and competitor algorithms in solving the CEC 2017 test suite is presented as boxplot diagrams in Fig. 3. These diagrams intuitively show that STBO has performed more effectively in solving most of the benchmark functions of the CEC 2017 test suite by providing better results compared to competitor algorithms.

**Table 8:** Evaluation results on the CEC 2017 test suite functions.

		GA	PSO	GSA	TLBO	MVO	GWO	WOA	TSA	MPA	RSA	AVOA	STBO
C17-F1	Mean	1.00E+02	1.48E+03	1.32E+10	1.14E+05	2.87E+09	8.69E+06	9.43E+03	2.30E+05	7.67E+07	2.45E+02	3.33E+03	1.65E+07
	Best	1.00E+02	1.05E+02	9.16E+09	1.76E+02	3.93E+08	2.04E+06	4.43E+03	1.93E+04	5.38E+07	1.00E+02	7.03E+02	1.05E+07
	Worst	1.00E+02	3.34E+03	1.82E+10	4.52E+05	5.36E+09	2.43E+07	1.48E+04	6.81E+05	1.19E+08	5.29E+02	5.63E+03	2.48E+07
	Std	1.02E−05	1.38E+03	3.95E+09	2.30E+05	2.21E+09	1.07E+07	4.47E+03	3.18E+05	3.04E+07	2.03E+02	2.73E+03	6.38E+06
	Median	1.00E+02	1.24E+03	1.27E+10	1.82E+03	2.87E+09	4.22E+06	9.23E+03	1.11E+05	6.69E+07	1.76E+02	3.49E+03	1.53E+07
	Rank	1	3	12	6	11	8	5	7	10	2	4	9
C17-F3	Mean	3.00E+02	3.65E+02	1.01E+04	3.39E+02	1.27E+04	3.54E+03	3.00E+02	4.66E+03	7.63E+02	8.81E+03	3.00E+02	2.48E+04
	Best	3.00E+02	3.00E+02	6.88E+03	3.00E+02	9.43E+03	1.24E+03	3.00E+02	2.58E+03	5.86E+02	4.05E+03	3.00E+02	1.80E+04
	Worst	3.00E+02	4.17E+02	1.74E+04	3.95E+02	1.54E+04	7.10E+03	3.00E+02	8.46E+03	9.28E+02	1.50E+04	3.00E+02	4.00E+04
	Std	1.57E−10	4.91E+01	4.98E+03	4.48E+01	3.04E+03	2.66E+03	5.88E−02	2.76E+03	1.80E+02	4.65E+03	2.49E−12	1.04E+04
	Med	3.00E+02	3.72E+02	8.11E+03	3.30E+02	1.31E+04	2.91E+03	3.00E+02	3.80E+03	7.69E+02	8.11E+03	3.00E+02	2.06E+04
	Rank	2	5	10	4	11	7	3	8	6	9	1	12
C17-F4	Mean	4.00E+02	4.23E+02	1.09E+03	4.04E+02	6.38E+02	4.24E+02	4.05E+02	4.18E+02	4.13E+02	4.07E+02	4.07E+02	4.16E+02
	Best	4.00E+02	4.00E+02	6.81E+02	4.00E+02	4.08E+02	4.08E+02	4.04E+02	4.07E+02	4.10E+02	4.07E+02	4.01E+02	4.13E+02
	Worst	4.00E+02	4.79E+02	1.92E+03	4.06E+02	1.09E+03	4.41E+02	4.06E+02	4.39E+02	4.20E+02	4.07E+02	4.11E+02	4.24E+02
	Std	7.08E−09	3.83E+01	5.78E+02	2.78E+00	3.25E+02	1.83E+01	9.96E−01	1.52E+01	5.12E+00	1.79E−01	4.35E+00	5.41E+00
	Median	4.00E+02	4.06E+02	8.77E+02	4.05E+02	5.26E+02	4.22E+02	4.05E+02	4.12E+02	4.11E+02	4.07E+02	4.08E+02	4.14E+02
	Rank	1	9	12	2	11	10	3	8	6	4	5	7
C17-F5	Mean	5.09E+02	5.43E+02	5.71E+02	5.20E+02	5.55E+02	5.57E+02	5.17E+02	5.15E+02	5.39E+02	5.48E+02	5.39E+02	5.32E+02
	Best	5.08E+02	5.36E+02	5.61E+02	5.12E+02	5.26E+02	5.30E+02	5.11E+02	5.09E+02	5.31E+02	5.37E+02	5.24E+02	5.27E+02
	Worst	5.11E+02	5.62E+02	5.90E+02	5.24E+02	5.91E+02	5.96E+02	5.23E+02	5.20E+02	5.50E+02	5.62E+02	5.72E+02	5.38E+02
	Std	1.31E+00	1.28E+01	1.34E+01	5.77E+00	3.20E+01	2.94E+01	5.24E+00	4.78E+00	7.78E+00	1.10E+01	2.24E+01	4.70E+00
	Med	5.09E+02	5.37E+02	5.67E+02	5.23E+02	5.52E+02	5.51E+02	5.17E+02	5.16E+02	5.38E+02	5.47E+02	5.31E+02	5.32E+02
	Rank	1	8	12	4	10	11	3	2	6	9	7	5
C17-F6	Mean	6.00E+02	6.21E+02	6.49E+02	6.00E+02	6.28E+02	6.32E+02	6.01E+02	6.01E+02	6.05E+02	6.25E+02	6.03E+02	6.08E+02
	Best	6.00E+02	6.11E+02	6.43E+02	6.00E+02	6.13E+02	6.17E+02	6.00E+02	6.00E+02	6.04E+02	6.14E+02	6.01E+02	6.05E+02
	Worst	6.00E+02	6.36E+02	6.56E+02	6.01E+02	6.48E+02	6.49E+02	6.02E+02	6.05E+02	6.07E+02	6.39E+02	6.06E+02	6.11E+02
	Std	3.09E−04	1.17E+01	5.42E+00	6.82E−01	1.61E+01	1.52E+01	7.69E−01	2.33E+00	1.33E+00	1.07E+01	2.33E+00	3.41E+00
	Median	6.00E+02	6.19E+02	6.49E+02	6.00E+02	6.26E+02	6.31E+02	6.01E+02	6.00E+02	6.05E+02	6.24E+02	6.03E+02	6.08E+02
	Rank	1	8	12	2	10	11	3	4	6	9	5	7
C17-F7	Mean	7.22E+02	7.65E+02	8.07E+02	7.26E+02	7.92E+02	7.93E+02	7.28E+02	7.41E+02	7.59E+02	7.18E+02	7.46E+02	7.37E+02
	Best	7.19E+02	7.51E+02	7.97E+02	7.14E+02	7.69E+02	7.66E+02	7.23E+02	7.32E+02	7.55E+02	7.14E+02	7.31E+02	7.29E+02
	Worst	7.24E+02	7.82E+02	8.12E+02	7.42E+02	8.23E+02	8.12E+02	7.37E+02	7.49E+02	7.66E+02	7.22E+02	7.76E+02	7.40E+02
	Std	2.01E+00	1.37E+01	7.26E+00	1.22E+01	2.42E+01	2.18E+01	6.17E+00	7.53E+00	4.73E+00	3.18E+00	2.14E+01	5.52E+00
	Med	7.22E+02	7.64E+02	8.09E+02	7.23E+02	7.89E+02	7.96E+02	7.26E+02	7.41E+02	7.57E+02	7.17E+02	7.39E+02	7.39E+02
	Rank	2	9	12	3	10	11	4	6	8	1	7	5
C17-F8	Mean	8.08E+02	8.29E+02	8.59E+02	8.10E+02	8.49E+02	8.33E+02	8.31E+02	8.16E+02	8.33E+02	8.19E+02	8.25E+02	8.23E+02
	Best	8.05E+02	8.21E+02	8.56E+02	8.07E+02	8.44E+02	8.14E+02	8.20E+02	8.13E+02	8.28E+02	8.16E+02	8.13E+02	8.17E+02
	Worst	8.10E+02	8.44E+02	8.62E+02	8.12E+02	8.55E+02	8.53E+02	8.62E+02	8.21E+02	8.37E+02	8.23E+02	8.39E+02	8.37E+02
	Std	2.25E+00	1.06E+01	3.14E+00	2.67E+00	5.22E+00	1.61E+01	2.11E+01	3.76E+00	4.03E+00	3.03E+00	1.13E+01	9.46E+00
	Median	8.09E+02	8.25E+02	8.58E+02	8.10E+02	8.49E+02	8.32E+02	8.21E+02	8.15E+02	8.35E+02	8.19E+02	8.24E+02	8.20E+02
	Rank	1	7	12	2	11	9	8	3	10	4	6	5
C17-F9	Mean	9.00E+02	1.35E+03	1.44E+03	9.26E+02	1.60E+03	1.56E+03	9.00E+02	9.01E+02	9.49E+02	9.00E+02	9.59E+02	9.05E+02
	Best	9.00E+02	9.42E+02	1.14E+03	9.01E+02	1.01E+03	1.04E+03	9.00E+02	9.00E+02	9.28E+02	9.00E+02	9.02E+02	9.02E+02
	Worst	9.00E+02	1.80E+03	1.85E+03	9.93E+02	2.52E+03	2.51E+03	9.01E+02	9.03E+02	9.89E+02	9.00E+02	1.03E+03	9.07E+02
	Std	2.65E−08	3.61E+02	3.04E+02	4.53E+01	7.29E+02	6.63E+02	4.44E−01	1.34E+00	2.76E+01	0.00E+00	5.22E+01	2.20E+00
	Med	9.00E+02	1.34E+03	1.38E+03	9.05E+02	1.43E+03	1.35E+03	9.00E+02	9.00E+02	9.39E+02	9.00E+02	9.54E+02	9.06E+02
	Rank	2	9	10	6	12	11	3	4	7	1	8	5
C17-F10	Mean	1.45E+03	2.25E+03	2.47E+03	1.84E+03	2.33E+03	2.25E+03	1.61E+03	1.71E+03	2.16E+03	2.67E+03	1.98E+03	1.78E+03
	Best	1.34E+03	1.92E+03	2.20E+03	1.12E+03	1.52E+03	1.89E+03	1.50E+03	1.61E+03	2.07E+03	2.23E+03	1.84E+03	1.53E+03
	Worst	1.61E+03	2.49E+03	2.78E+03	2.22E+03	2.74E+03	2.75E+03	1.78E+03	1.79E+03	2.23E+03	3.04E+03	2.28E+03	2.02E+03
	Std	1.22E+02	2.73E+02	2.78E+02	5.23E+02	5.64E+02	4.17E+02	1.34E+02	7.88E+01	8.62E+01	3.58E+02	2.11E+02	2.25E+02
	Median	1.42E+03	2.31E+03	2.46E+03	2.01E+03	2.53E+03	2.19E+03	1.59E+03	1.71E+03	2.16E+03	2.70E+03	1.90E+03	1.78E+03
	Rank	1	9	11	5	10	8	2	3	7	12	6	4
C17-F11	Mean	1.10E+03	1.25E+03	3.99E+03	1.12E+03	1.29E+03	1.22E+03	1.13E+03	1.14E+03	1.15E+03	1.17E+03	1.14E+03	5.16E+03
	Best	1.10E+03	1.17E+03	2.13E+03	1.11E+03	1.15E+03	1.12E+03	1.11E+03	1.13E+03	1.13E+03	1.13E+03	1.11E+03	1.36E+03
	Worst	1.10E+03	1.36E+03	5.72E+03	1.12E+03	1.49E+03	1.41E+03	1.14E+03	1.16E+03	1.17E+03	1.20E+03	1.15E+03	1.05E+04
	Std	1.11E+00	8.99E+01	1.76E+03	4.12E+00	1.44E+02	1.28E+02	1.49E+01	1.01E+01	1.77E+01	3.22E+01	1.71E+01	4.07E+03
	Med	1.10E+03	1.23E+03	4.05E+03	1.11E+03	1.26E+03	1.18E+03	1.12E+03	1.14E+03	1.15E+03	1.17E+03	1.14E+03	4.38E+03
	Rank	1	9	11	2	10	8	3	5	6	7	4	12
C17-F12	Mean	1.21E+03	1.24E+06	6.77E+07	3.04E+03	8.96E+07	9.35E+06	5.18E+05	1.52E+05	2.30E+06	8.96E+05	1.47E+04	1.69E+06
	Best	1.20E+03	3.82E+04	3.05E+07	1.67E+03	3.30E+05	5.80E+04	8.01E+03	4.19E+04	4.93E+05	9.83E+03	1.56E+03	1.72E+05
	Worst	1.24E+03	4.05E+06	1.04E+08	5.43E+03	3.54E+08	2.04E+07	1.29E+06	4.67E+05	3.64E+06	2.59E+06	2.45E+04	5.80E+06
	Std	1.84E+01	1.92E+06	3.39E+07	1.70E+03	1.80E+08	9.95E+06	6.30E+05	2.15E+05	1.45E+06	1.21E+06	1.02E+04	2.79E+06
	Median	1.20E+03	4.39E+05	6.79E+07	2.53E+03	1.92E+06	8.48E+06	3.84E+05	4.93E+04	2.53E+06	4.92E+05	1.64E+04	4.01E+05
	Rank	1	7	11	2	12	10	5	4	9	6	3	8
C17-F13	Mean	1.31E+03	1.22E+04	4.09E+07	1.34E+03	1.62E+04	2.04E+04	1.35E+04	1.18E+04	7.10E+03	1.25E+04	5.58E+03	7.05E+04
	Best	1.30E+03	4.63E+03	1.15E+05	1.31E+03	7.21E+03	8.27E+03	2.36E+03	7.43E+03	4.00E+03	7.36E+03	2.15E+03	1.22E+04
	Worst	1.31E+03	2.02E+04	1.23E+08	1.36E+03	2.19E+04	3.46E+04	2.77E+04	1.87E+04	1.17E+04	1.60E+04	9.59E+03	1.69E+05
	Std	3.93E+00	6.58E+03	5.78E+07	2.26E+01	7.12E+03	1.12E+04	1.07E+04	5.14E+03	3.46E+03	3.86E+03	3.12E+03	7.46E+04
	Med	1.31E+03	1.19E+04	2.05E+07	1.35E+03	1.79E+04	1.95E+04	1.20E+04	1.05E+04	6.35E+03	1.33E+04	5.29E+03	5.06E+04
	Rank	1	6	12	2	9	10	8	5	4	7	3	11
C17-F14	Mean	1.40E+03	2.55E+03	5.47E+03	1.43E+03	4.77E+03	1.58E+03	1.44E+03	2.83E+03	1.52E+03	5.57E+03	7.00E+03	6.76E+03
	Best	1.40E+03	2.01E+03	2.19E+03	1.40E+03	2.57E+03	1.50E+03	1.43E+03	1.48E+03	1.48E+03	2.07E+03	3.68E+03	1.87E+03
	Worst	1.40E+03	2.91E+03	8.08E+03	1.45E+03	5.60E+03	1.69E+03	1.44E+03	4.80E+03	1.56E+03	9.48E+03	1.15E+04	1.16E+04
	Std	1.92E+00	3.95E+02	2.57E+03	2.26E+01	1.49E+03	8.89E+01	5.16E+00	1.61E+03	3.77E+01	3.12E+03	3.32E+03	5.26E+03
	Median	1.40E+03	2.63E+03	5.80E+03	1.43E+03	5.44E+03	1.58E+03	1.44E+03	2.53E+03	1.53E+03	5.36E+03	6.43E+03	6.80E+03
	Rank	1	6	9	2	8	5	3	7	4	10	12	11
C17-F15	Mean	1.50E+03	5.27E+03	9.25E+03	1.51E+03	1.47E+04	5.77E+03	1.56E+03	5.41E+03	1.79E+03	1.54E+04	4.46E+03	4.36E+03
	Best	1.50E+03	1.95E+03	4.96E+03	1.50E+03	4.16E+03	2.03E+03	1.54E+03	1.81E+03	1.69E+03	6.55E+03	2.27E+03	1.87E+03
	Worst	1.50E+03	8.28E+03	1.65E+04	1.52E+03	2.43E+04	1.51E+04	1.58E+03	7.23E+03	2.01E+03	2.03E+04	6.87E+03	7.09E+03
	Std	6.65E−02	2.67E+03	5.30E+03	7.02E+00	1.11E+04	6.36E+03	1.87E+01	2.54E+03	1.51E+02	6.25E+03	1.95E+03	2.87E+03
	Med	1.50E+03	5.43E+03	7.77E+03	1.51E+03	1.52E+04	2.98E+03	1.56E+03	6.30E+03	1.73E+03	1.74E+04	4.35E+03	4.23E+03
	Rank	1	7	10	2	11	9	3	8	4	12	6	5
C17-F16	Mean	1.60E+03	1.84E+03	2.05E+03	1.69E+03	2.14E+03	1.86E+03	1.88E+03	1.75E+03	1.70E+03	2.21E+03	1.87E+03	1.82E+03
	Best	1.60E+03	1.75E+03	2.02E+03	1.60E+03	1.99E+03	1.66E+03	1.72E+03	1.61E+03	1.64E+03	2.16E+03	1.72E+03	1.75E+03
	Worst	1.60E+03	1.96E+03	2.07E+03	1.84E+03	2.37E+03	2.09E+03	2.03E+03	2.00E+03	1.86E+03	2.30E+03	1.97E+03	1.85E+03
	Std	2.75E−01	9.69E+01	2.05E+01	1.17E+02	1.74E+02	2.12E+02	1.28E+02	1.78E+02	1.05E+02	6.08E+01	1.20E+02	4.95E+01
	Median	1.60E+03	1.82E+03	2.05E+03	1.66E+03	2.10E+03	1.85E+03	1.88E+03	1.70E+03	1.66E+03	2.19E+03	1.90E+03	1.85E+03
	Rank	1	6	10	2	11	7	9	4	3	12	8	5
C17-F17	Mean	1.72E+03	1.76E+03	1.86E+03	1.74E+03	1.86E+03	1.87E+03	1.80E+03	1.77E+03	1.76E+03	1.97E+03	1.86E+03	1.75E+03
	Best	1.71E+03	1.72E+03	1.82E+03	1.73E+03	1.80E+03	1.82E+03	1.73E+03	1.74E+03	1.76E+03	1.76E+03	1.77E+03	1.75E+03
	Worst	1.72E+03	1.81E+03	1.93E+03	1.75E+03	1.97E+03	1.92E+03	1.86E+03	1.80E+03	1.76E+03	2.15E+03	1.98E+03	1.76E+03
	Std	7.86E+00	4.00E+01	5.17E+01	8.37E+00	7.34E+01	4.15E+01	6.32E+01	2.90E+01	1.03E+00	1.64E+02	9.71E+01	2.51E+00
	Med	1.72E+03	1.75E+03	1.85E+03	1.74E+03	1.84E+03	1.86E+03	1.80E+03	1.77E+03	1.76E+03	1.98E+03	1.85E+03	1.75E+03
	Rank	1	4	8	2	10	11	7	6	5	12	9	3
C17-F18	Mean	1.80E+03	2.35E+04	9.48E+07	1.83E+03	2.89E+04	2.46E+04	1.74E+04	2.31E+04	4.03E+04	1.20E+04	1.13E+04	9.29E+03
	Best	1.80E+03	8.83E+03	1.57E+06	1.81E+03	1.13E+04	3.35E+03	4.18E+03	7.85E+03	1.54E+04	8.16E+03	3.07E+03	4.53E+03
	Worst	1.80E+03	3.76E+04	3.69E+08	1.85E+03	3.77E+04	3.86E+04	3.82E+04	3.58E+04	5.63E+04	1.85E+04	1.74E+04	1.76E+04
	Std	5.82E−01	1.52E+04	1.87E+08	1.77E+01	1.25E+04	1.59E+04	1.66E+04	1.19E+04	1.80E+04	4.91E+03	6.76E+03	6.04E+03
	Median	1.80E+03	2.37E+04	4.22E+06	1.84E+03	3.34E+04	2.82E+04	1.36E+04	2.43E+04	4.48E+04	1.06E+04	1.24E+04	7.52E+03
	Rank	1	8	12	2	10	9	6	7	11	5	4	3
C17-F19	Mean	1.90E+03	9.13E+03	6.79E+05	1.91E+03	7.35E+04	2.93E+05	2.02E+03	6.14E+03	2.16E+03	2.89E+04	1.14E+04	2.01E+04
	Best	1.90E+03	3.26E+03	1.61E+05	1.90E+03	2.01E+03	7.90E+03	1.93E+03	1.93E+03	2.04E+03	8.74E+03	5.50E+03	8.22E+03
	Worst	1.90E+03	1.53E+04	1.87E+06	1.92E+03	2.76E+05	1.12E+06	2.27E+03	1.20E+04	2.36E+03	5.38E+04	2.00E+04	2.99E+04
	Std	6.62E−02	5.07E+03	8.20E+05	6.11E+00	1.38E+05	5.62E+05	1.71E+02	5.14E+03	1.46E+02	2.03E+04	6.42E+03	1.15E+04
	Med	1.90E+03	8.96E+03	3.41E+05	1.91E+03	8.08E+03	2.23E+04	1.95E+03	5.29E+03	2.11E+03	2.66E+04	1.01E+04	2.11E+04
	Rank	1	6	12	2	10	11	3	5	4	9	7	8
C17-F20	Mean	2.01E+03	2.12E+03	2.30E+03	2.02E+03	2.21E+03	2.26E+03	2.16E+03	2.05E+03	2.08E+03	2.33E+03	2.24E+03	2.05E+03
	Best	2.00E+03	2.03E+03	2.25E+03	2.02E+03	2.09E+03	2.07E+03	2.03E+03	2.03E+03	2.06E+03	2.20E+03	2.20E+03	2.04E+03
	Worst	2.02E+03	2.16E+03	2.36E+03	2.04E+03	2.43E+03	2.35E+03	2.26E+03	2.08E+03	2.12E+03	2.42E+03	2.27E+03	2.08E+03
	Std	1.04E+01	6.44E+01	5.25E+01	9.05E+00	1.52E+02	1.32E+02	9.92E+01	2.46E+01	2.61E+01	9.56E+01	3.57E+01	1.88E+01
	Median	2.01E+03	2.14E+03	2.30E+03	2.02E+03	2.17E+03	2.30E+03	2.17E+03	2.06E+03	2.08E+03	2.35E+03	2.24E+03	2.05E+03
	Rank	1	6	11	2	8	10	7	4	5	12	9	3
C17-F21	Mean	2.20E+03	2.30E+03	2.31E+03	2.29E+03	2.35E+03	2.34E+03	2.30E+03	2.29E+03	2.30E+03	2.36E+03	2.32E+03	2.31E+03
	Best	2.20E+03	2.20E+03	2.25E+03	2.21E+03	2.34E+03	2.32E+03	2.20E+03	2.20E+03	2.21E+03	2.36E+03	2.31E+03	2.22E+03
	Worst	2.20E+03	2.36E+03	2.39E+03	2.32E+03	2.38E+03	2.35E+03	2.34E+03	2.32E+03	2.34E+03	2.37E+03	2.34E+03	2.34E+03
	Std	1.24E−05	6.89E+01	6.02E+01	5.53E+01	1.93E+01	1.50E+01	6.49E+01	6.17E+01	6.81E+01	5.99E+00	1.16E+01	5.84E+01
	Med	2.20E+03	2.32E+03	2.30E+03	2.31E+03	2.34E+03	2.34E+03	2.33E+03	2.32E+03	2.34E+03	2.36E+03	2.32E+03	2.33E+03
	Rank	1	5	7	2	11	10	4	3	6	12	9	8
C17-F22	Mean	2.30E+03	2.31E+03	2.96E+03	2.31E+03	2.39E+03	2.32E+03	2.30E+03	2.31E+03	2.32E+03	2.30E+03	2.31E+03	2.32E+03
	Best	2.30E+03	2.30E+03	2.76E+03	2.30E+03	2.31E+03	2.31E+03	2.30E+03	2.30E+03	2.32E+03	2.30E+03	2.30E+03	2.32E+03
	Worst	2.30E+03	2.31E+03	3.25E+03	2.31E+03	2.47E+03	2.33E+03	2.30E+03	2.32E+03	2.33E+03	2.30E+03	2.35E+03	2.33E+03
	Std	4.86E−01	3.68E+00	2.15E+02	3.55E+00	9.17E+01	7.91E+00	9.78E−01	6.71E+00	7.38E+00	1.81E−01	2.38E+01	5.84E+00
	Median	2.30E+03	2.31E+03	2.92E+03	2.31E+03	2.39E+03	2.32E+03	2.30E+03	2.30E+03	2.33E+03	2.30E+03	2.30E+03	2.32E+03
	Rank	2	6	12	5	11	8	3	4	10	1	7	9
C17-F23	Mean	2.61E+03	2.67E+03	2.70E+03	2.65E+03	2.69E+03	2.66E+03	2.61E+03	2.62E+03	2.64E+03	2.73E+03	2.64E+03	2.66E+03
	Best	2.61E+03	2.65E+03	2.67E+03	2.62E+03	2.67E+03	2.61E+03	2.61E+03	2.61E+03	2.62E+03	2.72E+03	2.61E+03	2.65E+03
	Worst	2.61E+03	2.69E+03	2.72E+03	2.67E+03	2.72E+03	2.69E+03	2.62E+03	2.64E+03	2.65E+03	2.75E+03	2.66E+03	2.68E+03
	Std	2.10E+00	1.90E+01	2.05E+01	2.11E+01	1.81E+01	3.45E+01	6.24E+00	1.63E+01	1.11E+01	1.00E+01	2.06E+01	1.47E+01
	Med	2.61E+03	2.67E+03	2.70E+03	2.65E+03	2.69E+03	2.66E+03	2.61E+03	2.62E+03	2.64E+03	2.73E+03	2.65E+03	2.66E+03
	Rank	1	9	11	6	10	7	2	3	4	12	5	8
C17-F24	Mean	2.50E+03	2.78E+03	2.89E+03	2.64E+03	2.82E+03	2.80E+03	2.75E+03	2.75E+03	2.77E+03	2.74E+03	2.78E+03	2.77E+03
	Best	2.50E+03	2.76E+03	2.84E+03	2.50E+03	2.80E+03	2.75E+03	2.75E+03	2.74E+03	2.76E+03	2.50E+03	2.77E+03	2.77E+03
	Worst	2.50E+03	2.80E+03	2.93E+03	2.78E+03	2.85E+03	2.82E+03	2.76E+03	2.78E+03	2.77E+03	2.86E+03	2.78E+03	2.79E+03
	Std	4.88E−05	1.46E+01	3.77E+01	1.66E+02	2.29E+01	3.01E+01	4.03E+00	2.06E+01	5.94E+00	1.66E+02	3.92E+00	1.05E+01
	Median	2.50E+03	2.77E+03	2.90E+03	2.64E+03	2.82E+03	2.81E+03	2.75E+03	2.74E+03	2.77E+03	2.80E+03	2.78E+03	2.77E+03
	Rank	1	8	12	2	11	10	5	4	6	3	9	7
C17-F25	Mean	2.90E+03	2.94E+03	3.38E+03	2.93E+03	3.13E+03	2.95E+03	2.90E+03	2.94E+03	2.93E+03	2.93E+03	2.93E+03	2.95E+03
	Best	2.90E+03	2.90E+03	3.35E+03	2.90E+03	2.94E+03	2.95E+03	2.90E+03	2.92E+03	2.91E+03	2.90E+03	2.90E+03	2.95E+03
	Worst	2.90E+03	2.95E+03	3.46E+03	2.95E+03	3.50E+03	2.96E+03	2.90E+03	2.95E+03	2.95E+03	2.94E+03	2.95E+03	2.96E+03
	Std	3.10E−07	2.38E+01	5.00E+01	2.38E+01	2.59E+02	5.22E+00	2.51E−01	1.57E+01	1.63E+01	2.24E+01	2.41E+01	2.74E+00
	Med	2.90E+03	2.95E+03	3.36E+03	2.95E+03	3.03E+03	2.95E+03	2.90E+03	2.95E+03	2.93E+03	2.94E+03	2.95E+03	2.95E+03
	Rank	1	7	12	5	11	9	2	8	3	4	6	10
C17-F26	Mean	2.88E+03	2.97E+03	4.24E+03	3.26E+03	3.88E+03	3.26E+03	2.90E+03	2.96E+03	3.29E+03	4.13E+03	2.85E+03	3.02E+03
	Best	2.80E+03	2.82E+03	3.82E+03	2.90E+03	2.91E+03	2.83E+03	2.90E+03	2.90E+03	2.99E+03	3.57E+03	2.60E+03	2.91E+03
	Worst	2.90E+03	3.14E+03	4.79E+03	3.96E+03	4.77E+03	3.97E+03	2.90E+03	2.98E+03	4.17E+03	4.43E+03	3.02E+03	3.13E+03
	Std	5.10E+01	1.84E+02	4.62E+02	4.81E+02	7.79E+02	5.06E+02	4.02E−02	3.84E+01	5.98E+02	3.89E+02	1.98E+02	1.01E+02
	Median	2.90E+03	2.97E+03	4.17E+03	3.10E+03	3.93E+03	3.12E+03	2.90E+03	2.97E+03	3.00E+03	4.25E+03	2.89E+03	3.03E+03
	Rank	2	5	12	8	10	7	3	4	9	11	1	6
C17-F27	Mean	3.09E+03	3.10E+03	3.17E+03	3.11E+03	3.17E+03	3.17E+03	3.09E+03	3.09E+03	3.11E+03	3.30E+03	3.12E+03	3.15E+03
	Best	3.09E+03	3.10E+03	3.14E+03	3.10E+03	3.14E+03	3.13E+03	3.09E+03	3.09E+03	3.09E+03	3.22E+03	3.10E+03	3.13E+03
	Worst	3.09E+03	3.10E+03	3.23E+03	3.13E+03	3.20E+03	3.21E+03	3.10E+03	3.09E+03	3.15E+03	3.38E+03	3.14E+03	3.18E+03
	Std	2.28E−01	3.21E+00	4.20E+01	1.62E+01	3.29E+01	3.43E+01	3.14E+00	2.68E+00	3.02E+01	7.96E+01	1.92E+01	1.79E+01
	Med	3.09E+03	3.10E+03	3.17E+03	3.11E+03	3.17E+03	3.18E+03	3.09E+03	3.09E+03	3.10E+03	3.29E+03	3.11E+03	3.15E+03
	Rank	1	4	11	6	9	10	3	2	5	12	7	8
C17-F28	Mean	3.03E+03	3.33E+03	3.91E+03	3.31E+03	3.47E+03	3.31E+03	3.33E+03	3.24E+03	3.44E+03	3.47E+03	3.32E+03	3.20E+03
	Best	2.80E+03	3.10E+03	3.87E+03	3.10E+03	3.40E+03	3.19E+03	3.15E+03	3.18E+03	3.23E+03	3.42E+03	3.18E+03	3.17E+03
	Worst	3.10E+03	3.41E+03	3.95E+03	3.44E+03	3.60E+03	3.41E+03	3.41E+03	3.40E+03	3.73E+03	3.52E+03	3.41E+03	3.21E+03
	Std	1.53E+02	1.59E+02	3.76E+01	1.54E+02	9.20E+01	1.23E+02	1.25E+02	1.07E+02	2.16E+02	4.14E+01	1.05E+02	2.22E+01
	Median	3.10E+03	3.41E+03	3.91E+03	3.34E+03	3.44E+03	3.32E+03	3.38E+03	3.20E+03	3.40E+03	3.47E+03	3.34E+03	3.20E+03
	Rank	1	8	12	4	11	5	7	3	9	10	6	2
C17-F29	Mean	3.14E+03	3.38E+03	3.34E+03	3.17E+03	3.31E+03	3.32E+03	3.23E+03	3.19E+03	3.23E+03	3.52E+03	3.29E+03	3.28E+03
	Best	3.13E+03	3.30E+03	3.21E+03	3.15E+03	3.29E+03	3.26E+03	3.18E+03	3.17E+03	3.18E+03	3.33E+03	3.21E+03	3.22E+03
	Worst	3.15E+03	3.44E+03	3.46E+03	3.19E+03	3.34E+03	3.40E+03	3.30E+03	3.20E+03	3.32E+03	3.70E+03	3.34E+03	3.34E+03
	Std	8.71E+00	6.37E+01	1.17E+02	1.68E+01	2.48E+01	6.04E+01	5.29E+01	1.18E+01	6.16E+01	1.64E+02	6.07E+01	5.18E+01
	Med	3.15E+03	3.38E+03	3.35E+03	3.16E+03	3.31E+03	3.32E+03	3.22E+03	3.19E+03	3.22E+03	3.52E+03	3.30E+03	3.27E+03
	Rank	1	11	10	2	8	9	4	3	5	12	7	6
C17-F30	Mean	3.41E+03	5.29E+05	5.68E+06	5.60E+03	4.72E+06	3.20E+06	3.79E+05	8.36E+05	3.11E+04	1.81E+06	6.31E+05	2.25E+06
	Best	3.40E+03	1.05E+04	9.84E+05	3.64E+03	2.49E+06	2.86E+04	1.47E+04	8.10E+03	2.12E+04	3.06E+05	3.87E+03	2.28E+05
	Worst	3.43E+03	1.20E+06	1.85E+07	1.10E+04	8.48E+06	6.05E+06	1.47E+06	1.70E+06	4.45E+04	5.57E+06	1.85E+06	4.21E+06
	Std	1.83E+01	5.90E+05	8.71E+06	3.70E+03	2.79E+06	2.95E+06	7.39E+05	9.73E+05	1.09E+04	2.57E+06	8.87E+05	2.04E+06
	Median	3.40E+03	4.52E+05	1.63E+06	3.87E+03	3.95E+06	3.37E+06	1.77E+04	8.19E+05	2.94E+04	6.93E+05	3.34E+05	2.29E+06
	Rank	1	5	12	2	11	10	4	7	3	8	6	9
Sum rank		34	200	320	96	298	261	125	141	181	228	177	201
Mean rank		1.1724	6.8965	11.0344	3.3103	10.2758	9	4.3103	4.8620	6.2413	7.8620	6.1034	6.9310
Total rank		1	7	12	2	11	10	3	4	6	9	5	8
*P*-value			6.882E−21	1.972E−21	1.289E−19	1.972E−21	1.972E−21	3.406E−20	3.881E−21	1.972E−21	1.803E−20	7.408E−20	1.972E−21

### STBO for real-world applications

**Figure 4:**
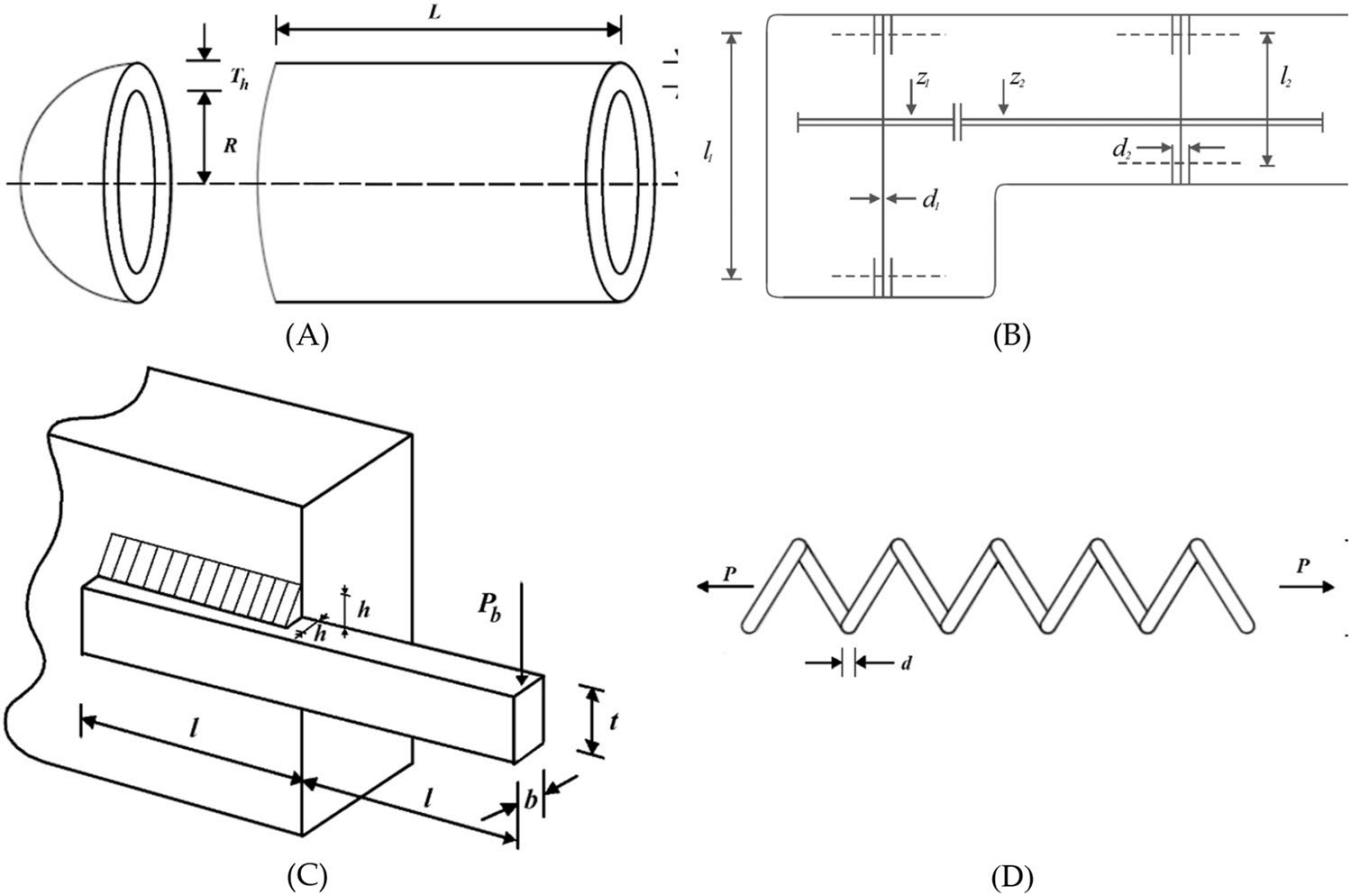
Schematics of four real-world applications: (A) PVD, (B) SRD, (C) WBD, (D) TCSD.

STBO's ability to optimize optimization problems in real-world applications is evaluated in this section. To this end, STBO and competitor algorithms have been implemented on four engineering optimization challenges. These engineering challenges are pressure vessel design (PVD)^79^, speed reducer design (SRD)^80^, welded beam design (WBD)^12^, and tension/compression spring design (TCSD)^12^. Schematics of these problems are presented in Fig. 4.

**Figure 5:**
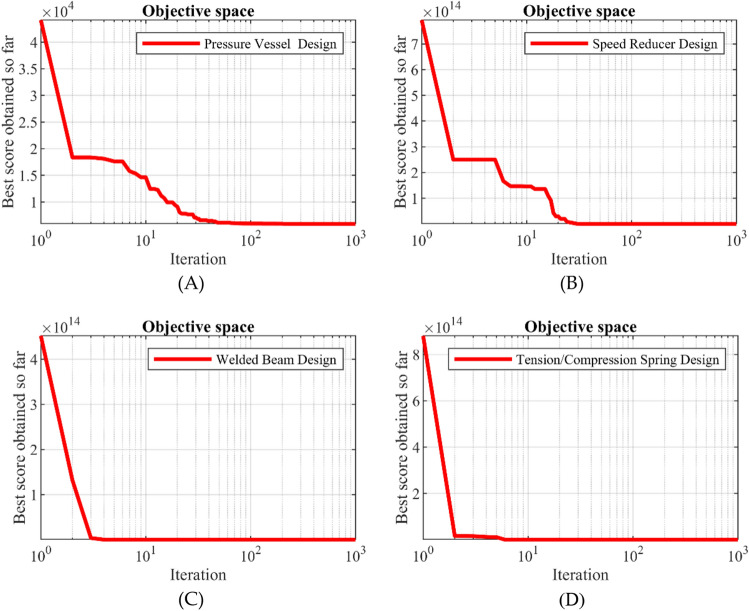
Convergence curves of STBO on four real-world applications.

The optimization results of the four mentioned challenges are reported in Table 9. The simulation results show that STBO performs superior to competitor algorithms in optimizing all four studied engineering challenges. What is clear from the analysis of the simulation results is that STBO has an effective capability in dealing with real-world optimization applications. The convergence curves of STBO while optimizing the mentioned optimization challenges are presented in Fig. 5. The convergence curves show that STBO has identified the main optimal area in the initial iterations by providing a desirable global search. Then, by increasing the iterations of the algorithm based on the local search, it tries to get better solutions. Intuitive analysis of convergence curves shows that STBO has converged to suitable solutions with a high ability to balance exploration and exploitation.

**Table 9:** Evaluation results of four real-world applications.

		GA	PSO	GSA	TLBO	MVO	GWO	WOA	TSA	MPA	RSA	AVOA	STBO
PVD	Mean	6645.562	6265.49	6842.164	6328.261	6478.841	6066.455	5892.921	5888.84	6117.763	6038.652	5963.405	5888.170
	Best	6581.043	5918.224	11,605	6166.438	6039.984	5919.289	5917.261	5913.451	6109.88	6031.364	5958.117	5884.882
	Worst	8007.337	7007.411	7160.988	6513.898	7252.635	7396.34	5896.021	5893.718	6129.069	6042.513	5968.94	5895.379
	Std	657.679	496.2457	5791.998	126.639	327.0846	66.63439	13.91331	28.93686	38.24161	31.18698	27.451658	23.71639
	Median	7587.808	6114.139	6839.254	6319.815	6398.997	6417.635	5892.046	5887.624	6115.578	6036.744	5962.3195	5887.907
	Rank	11	8	12	9	10	6	3	2	7	5	4	1
SRD	Mean	3190.666	3174.457	3069.904	3032.78	3109.29	3009.754	3003.541	3011.73	3001.864	3000.171	3000.197	3000.029
	Best	3070.629	3054.173	3033.594	3005.931	3008.769	3004.291	3001.55	3004.837	2996.216	2996.171	2995.7775	2995.39
	Worst	3317.508	3368.247	3108.816	3064.938	3215.349	3012.665	3007.795	3027.316	3007.093	3002.173	3001.897	3001.627
	Std	17.14086	92.69298	18.0977	13.03553	79.74166	5.845531	1.934443	10.36808	5.219098	2.015032	1.8193737	1.623719
	Med	3202.346	3160.857	3069.595	3030.968	3109.29	3008.426	3003.087	3010.34	3000.431	2999.836	2999.4455	2999.061
	Rank	12	11	9	8	10	6	5	7	4	2	3	1
WBD	Mean	1.96595	2.123005	2.54876	1.820886	1.732754	2.234273	1.730198	1.728896	1.892096	1.725025	1.7248133	1.724605
	Best	1.83841	1.876176	2.175414	1.761242	1.727502	1.822536	1.729027	1.727691	1.866157	1.727296	1.7252098	1.723127
	Worst	2.038864	2.324247	3.008994	1.876738	1.744746	3.053648	1.730634	1.729132	2.016418	1.727726	1.7272073	1.726692
	Std	0.139733	0.034882	0.256314	0.027592	0.004875	0.325102	0.001159	0.000287	0.00796	0.005124	0.004724	0.004324
	Median	1.939188	2.100775	2.499548	1.823362	1.73049	2.248652	1.730157	1.728855	1.883578	1.725997	1.7249368	1.72388
	Rank	9	10	12	7	6	11	5	4	8	3	2	1
TCSD	Mean	0.013192	0.014166	0.013564	0.01296	0.014599	0.014956	0.012816	0.012803	0.013898	0.0128	0.012737	0.012674
	Best	0.012889	0.013151	0.012987	0.012822	0.01293	0.013309	0.01279	0.012786	0.013218	0.012768	0.01271	0.012652
	Worst	0.015356	0.016403	0.014345	0.01312	0.018006	0.018029	0.01284	0.012834	0.01583	0.012812	0.0127475	0.012683
	Std	0.000378	0.002092	0.000289	0.007831	0.001637	0.002293	0.004193	0.005671	0.006141	0.007417	0.004219	0.001021
	Med	0.013073	0.013123	0.013492	0.012965	0.014151	0.013316	0.012819	0.012806	0.013776	0.01279	0.0127305	0.012671
	Rank	7	10	8	6	11	12	5	4	9	3	2	1
Sum rank		39	39	41	30	37	35	18	17	28	13	11	4
Mean rank		9.75	9.75	10.25	7.5	9.25	8.75	4.5	4.25	7	3.25	2.75	1
Total rank		8	11	12	7	10	9	5	4	6	3	2	1

### Ethical approval

This article does not contain any studies with human participants or animals performed by any of the authors.

### Informed consent

Informed consent was not required as no human or animals were involved.

## Conclusion and future works

This paper introduced a new metaheuristic algorithm called Sewing Training-Based Optimization (STBO) to solve optimization problems. The interactions between the training instructor and the beginner tailors are the main inspiration in the design of STBO. The proposed STBO was modeled and designed in three phases: (i) training, (ii) imitation of the instructor's skills, and (iii) practice. The STBO’s performance was tested on fifty-two objective functions of unimodal, high-dimensional multimodal, fixed-dimensional multimodal, and the CEC 2017 test suite. The optimization results of the benchmark functions showed that the proposed STBO approach has a good ability in exploration, exploitation, and balancing their proportion during the search process in the problem-solving space. Eleven well-known metaheuristic algorithms were employed to compare the performance of STBO in optimization. The simulation results showed that STBO has superior and competitive performance compared to some well-known metaheuristic algorithms, providing better results in most of the objective functions studied in this paper. STBO implementation on four engineering design challenges demonstrated the capability of the proposed algorithm in real-world applications.

Although the proposed STBO has provided good performance in most of the benchmark functions studied in this article, the proposed algorithm has some limitations. The first limitation of STBO is that it is always possible to devise newer algorithms that perform better than the proposed approach. The second limitation of STBO is that there is a possibility that the implementation of the proposed algorithm will fail in some optimization applications. Finally, the third limitation of STBO is that there is no guarantee that STBO can always provide a globally optimal solution since the proposed algorithm is based on a random search. Also, based on the concept of the NFL theorem, it is not claimed that STBO is the best optimizer for all optimization applications.

Introducing the STBO activates several research tasks for future studies. Developing binary and multimodal versions is a possible specific STBO research proposal. Employing STBO in various applications of optimization in science as well as in real-world applications are other suggestions for further studies.

## Data Availability

All data generated or analyzed during this study are included directly in the text of this submitted manuscript. There are no additional external files with datasets.
